# Induction of functional xeno-free MSCs from human iPSCs via a neural crest cell lineage

**DOI:** 10.1038/s41536-022-00241-8

**Published:** 2022-09-15

**Authors:** Daisuke Kamiya, Nana Takenaka-Ninagawa, Souta Motoike, Mikihito Kajiya, Teppei Akaboshi, Chengzhu Zhao, Mitsuaki Shibata, Sho Senda, Yayoi Toyooka, Hidetoshi Sakurai, Hidemi Kurihara, Makoto Ikeya

**Affiliations:** 1 Dept. of Clinical Application, Center for iPS Cell Research and Application (CiRA), Kyoto University, 53 Kawahara-cho, Shogoin, Sakyo-ku, Kyoto, 606-8507 Japan; 2 Takeda-CiRA Joint Program, Fujisawa, Kanagawa Japan; 3 Institute of Biomedical & Health Sciences, Graduate School of Biomedical & Health Sciences, Hiroshima University, 1-2-3, Kasumi, Minami-ku, Hiroshima, 734-8553 Japan; 4 Research Institute for Bioscience Product & Fine Chemicals, Ajinomoto Co., Inc., Kawasaki, 210-8681 Japan

**Keywords:** Mesenchymal stem cells, Stem-cell research, Regeneration

## Abstract

Mesenchymal stem/stromal cells (MSCs) are adult multipotent stem cells. Here, we induced MSCs from human induced pluripotent stem cells (iPSCs) via a neural crest cell (NCC) lineage under xeno-free conditions and evaluated their in vivo functions. We modified a previous MSC induction method to work under xeno-free conditions. Bovine serum albumin-containing NCC induction medium and fetal bovine serum-containing MSC induction medium were replaced with xeno-free medium. Through our optimized method, iPSCs differentiated into MSCs with high efficiency. To evaluate their in vivo activities, we transplanted the xeno-free-induced MSCs (XF-iMSCs) into mouse models for bone and skeletal muscle regeneration and confirmed their regenerative potency. These XF-iMSCs mainly promoted the regeneration of surrounding host cells, suggesting that they secrete soluble factors into affected regions. We also found that the peroxidasin and IGF2 secreted by the XF-iMSCs partially contributed to myotube differentiation. These results suggest that XF-iMSCs are important for future applications in regenerative medicine.

## Introduction

Mesenchymal stem/stromal cells (MSCs) are a cell population capable of differentiating into osteocytes, chondrocytes, and adipocytes in vitro and are present in several tissues, such as bone marrow, adipose tissue, synovium, dental pulp, and cord blood^1,2^. Bone marrow-derived MSCs have long been used for hematopoietic stem cell transplantation; hence, they are considered safe for medical use^3^. Currently, primary MSCs are used to treat several clinical complications, including graft-versus-host disease, Crohn’s disease^4^, ischemic cardiomyopathy^5^, and stroke^6^. These broad applications illustrate the significance of MSCs in cell therapy^7^.

The quality of MSCs varies depending on, but is not limited to, the donor’s health and medication intake. In addition, the number of MSCs obtained from elderly people is usually low because MSC production decreases in vivo with age^8^. Hence, the development of a method that provides a stable supply of high-quality MSCs is essential for cell therapy. Although various efforts have been made in this direction (such as the improvement of culture medium, optimization of oxygen conditions), such a method has not yet been developed.

Our research has focused on the development of a method for inducing MSCs from pluripotent stem cells^9^. Developmentally, MSCs are differentiated via mesodermal cells or neural crest cells (NCCs)^10–12^. NCCs are multipotent ectodermal cells that develop from the intermediate of the neuroectoderm and epidermal ectoderm during vertebrate development^13^. NCCs are known to differentiate into ectoderm lineage cells, such as peripheral nerve cells and glial cells; mesoderm cells, such as the bone cells, cartilage cells, and adipocytes through MSCs; and endoderm cells, such as hepatocytes and pancreatic cells. Based on this knowledge, we developed an easy and robust method to induce MSCs from human induced pluripotent stem cells (iPSCs) via NCCs^9^. Using this induction method, we conducted pathological analysis and drug discovery research on fibrodysplasia ossificans progressiva, a rare and refractory bone disease characterized by heterotopic ossification in muscle tissue^14–17^. However, this method contains animal-derived components, which are disadvantageous for cell therapy^18,19^.

In the present study, in continuation of our research and considering the future of regenerative medicine, we adapted our induction protocol to include xeno-free conditions and demonstrate that induced MSCs stimulate skeletal muscle and bone regeneration. This study may provide novel clinical treatments for intractable musculoskeletal disorders, such as osteoporosis and sarcopenia.

## Results

### Induction of NCCs from human iPSCs under xeno-free conditions

**Fig. 1:**
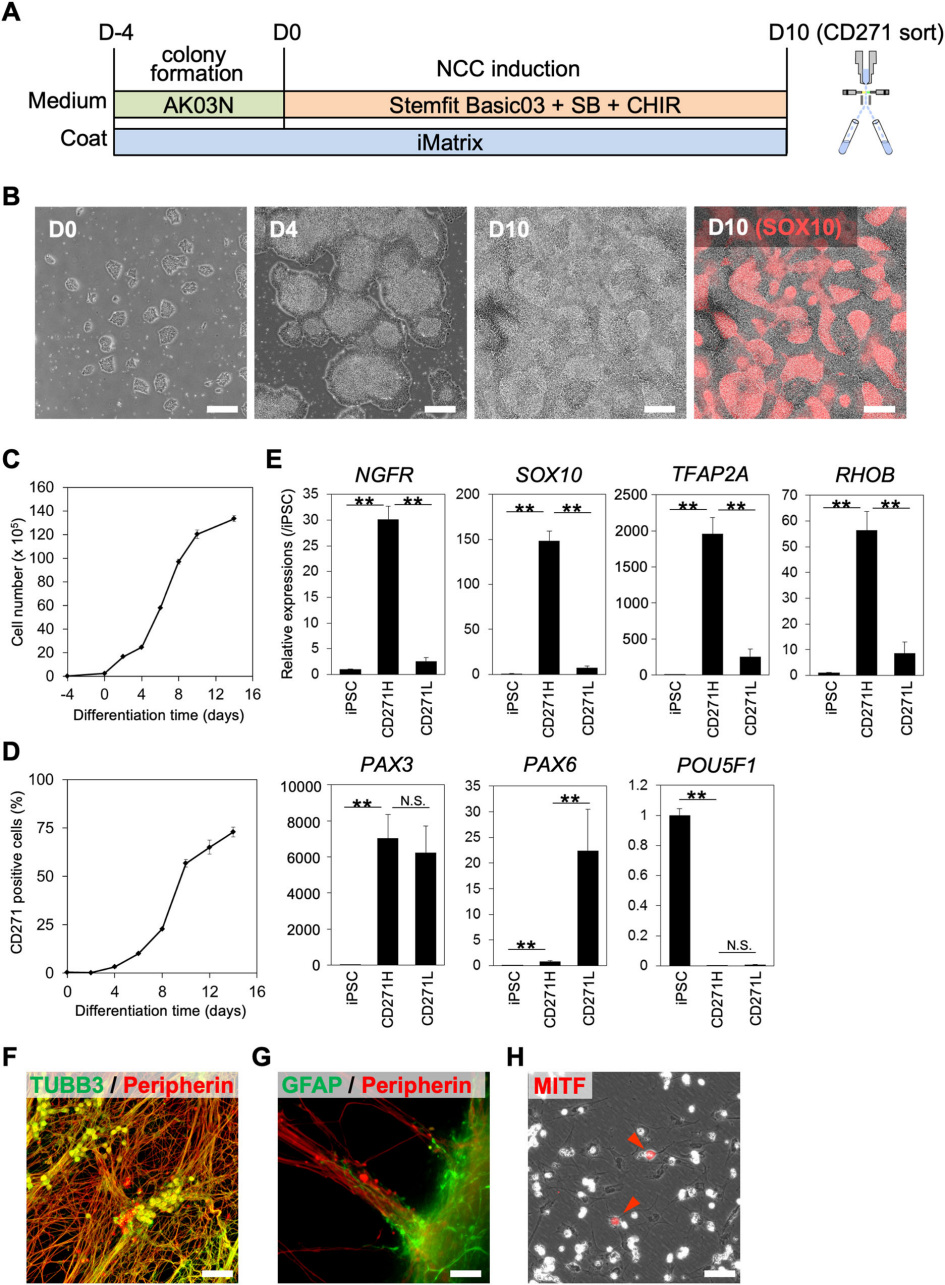
Efficient induction of iNCCs from iPSCs under xeno-free conditions. **A** Schematic representation of a neural crest induction protocol using AK03N and Basic03 xeno-free medium. **B** Morphology of colonies during the induction. Phase contrast images obtained on days 0, 4, and 10. In the far right panel, SOX10 (red color) expression was merged at day 10. Scale bars, 200 µm. **C** Numbers of cells during neural crest induction. Data are the mean ± SD, *n* = 3. **D** Percentage of CD271-positive cells during neural crest induction, as analyzed by FCM. Data are the mean ± SD, *n* = 3. **E** The expression of marker genes in sorted CD271^high^ (CD271H) and CD271^low^ (CD271L) cells. The mRNA expression of each gene was analyzed by RT-qPCR in 1231A3 iPSCs and CD271H and CD271L cells and is shown relative to the expression level in 1231A3 iPSCs. Data are the mean ± SD, *n* = 3. **P < 0.01. Neuronal cell (**F**), glial cell (**G**), and melanocyte (**H**) differentiation from CD271H-sorted cells. **F** Cells were stained with an anti-TUBB3 antibody (green) and anti-peripherin antibody (red). **G** Cells were stained with an anti-GFAP antibody (green) and anti-peripherin antibody (red). **H** Cells were stained with an anti-MITF antibody (red). Scale bars, 50 µm (**F**–**H**).

To induce NCCs from human iPSCs under xeno-free conditions, we modified the previous induction protocol. In the original protocol, we used iPSC lines maintained on an STO cell line transformed with neomycin resistance and LIF genes (SNL) stromal feeder cells in a culture dish coated with growth factor-reduced Matrigel, which was extracted from the Engelbreth–Holm–Swarm mouse sarcoma cell line. To avoid these animal components, we used a feeder-free and xeno-free iPSC line—1231A3 iPSCs—and maintained it in iMatrix (laminin-511 E8 fragment)-coated culture dishes and in Stemfit AK03N xeno-free (defined and animal component-free) culture media^20^ (Fig. 1A). We cultured the iPSCs for 4 days until colonies formed, before starting the NCC induction. In the original protocol, bovine serum albumin (BSA) was used during the NCC induction. Here, we replaced BSA-containing basal medium with Stemfit Basic03, which is comparable to AK03N minus bFGF. Ten days after the NCC induction in Stemfit Basic03 medium supplemented with 10 μM SB431542 (SB) and 1 μM CHIR99021 (CHIR), the cells nearly reached confluence (Fig. 1B, C) in the cell-dense region and expressed the NCC markers SOX10, CD271, and TFAP2A (Fig. 1B and Supplementary Fig. 1A). We also confirmed that SOX10 and CD271, a cell-surface marker of NCC, mostly overlapped. The induction efficiency was analyzed using fluorescence-activated cell sorting (FACS) with CD271 and SSEA4 antibodies. SSEA4-positive undifferentiated iPSCs were seldom detected (<0.05%) and the ratio of CD271^high^-positive cells reached a peak of 90% (Supplementary Fig. 1B, C). The robustness of this protocol was confirmed by repeated experiments using 1231A3 and other feeder-free and xeno-free iPSC lines (1231A3, 1381A5, 1381B5, 1383D2, and 1383D10; 71.8 ± 18.3%, 59.0 ± 13.7%, 55.2 ± 17.5%, 15.0 ± 11.1%, and 50.3 ± 13.3%, respectively) (Supplementary Fig. 1D). CD271^high^-positive cells emerged until day 4 and gradually increased during induction (Fig. 1D). Since CD271 recognizes the cell-surface protein NGFR (p75), cell sorting with the CD271 antibody allowed for enrichment of NCCs (Supplementary Fig. 1E); the enrichment was confirmed by the higher expression of NCC markers (*NGFR, SOX10, TFAP2A*, and *RHOB*) in CD271^high^ cells compared with that in the CD271^low^ cells (Fig. 1E). We also checked the expression of the neural plate border marker *PAX3* and neuroectoderm marker *PAX6* as well as the pluripotent cell marker *POUF5F1* using quantitative real-time polymerase chain reaction (RT-qPCR). We found that CD271^low^ cells included neural cells (Fig. 1E). The multiple differentiation potential of CD271^high^ cells (positive for TUBB3 and negative for Peripherin, GFAP and MITF) (Supplementary Fig. 2A) was confirmed by the induction of peripheral nervous system cells (TUBB3, Peripherin, and GFAP) (Fig. 1F, G) and melanocytes (MITF) (Fig. 1H). From CD271^low^ cells TUBB3-positive neurons were induced, but Peripherin-positive peripheral neurons were hardly detected, and MITF-positive pigment cells were not at all, consistent with the result that CD271^low^ cells included neuroectoderm (Supplementary Fig. 2B).

**Fig. 2:**
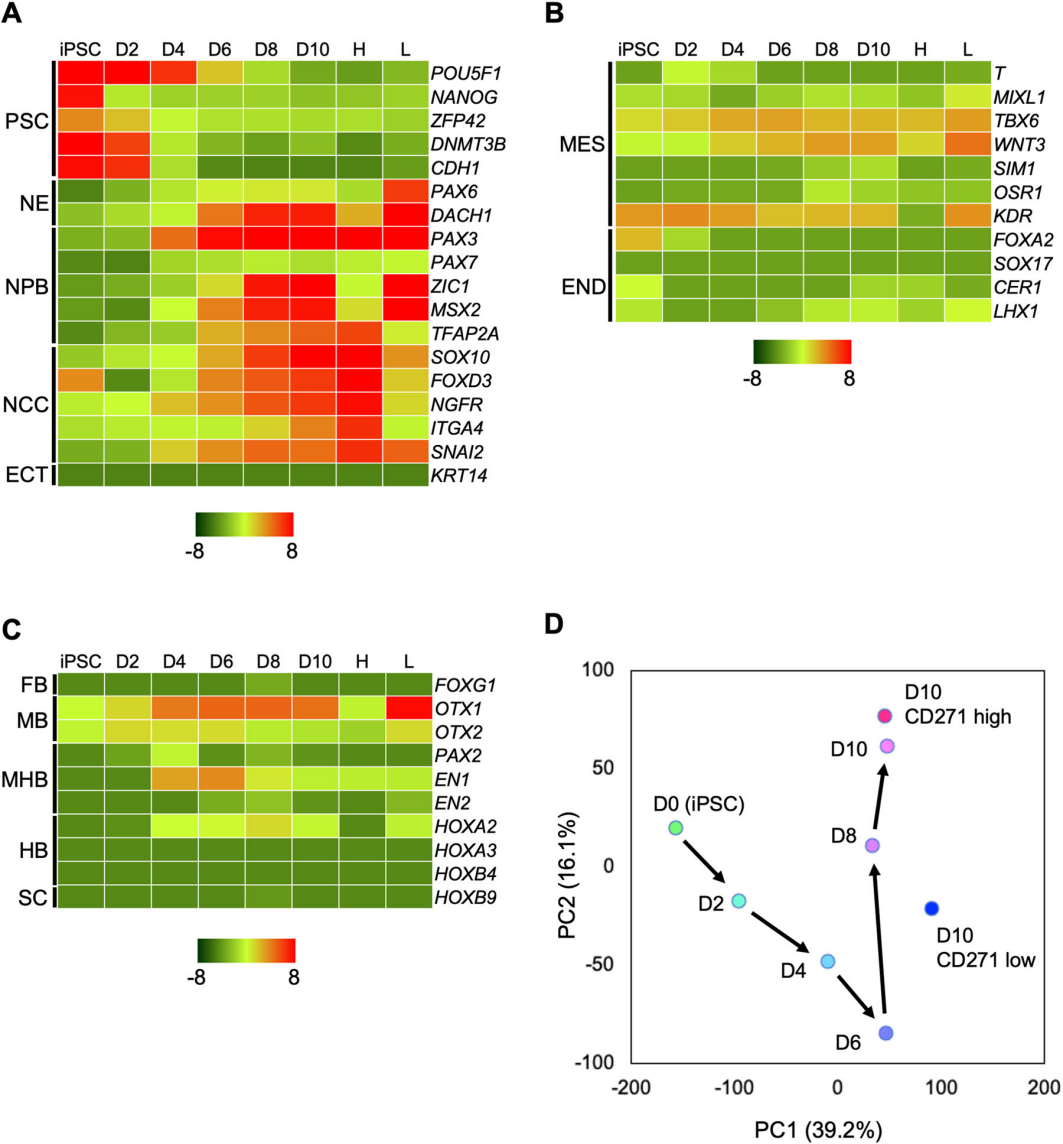
Global gene expression profile reveals the stepwise differentiation of iNCCs from iPSCs through ectodermal lineage. **A** A heatmap illustrating the expression of pluripotent stem cell (PSC), neuroectoderm (NE), neural plate border (NPB), neural crest cell (NCC), and ectoderm (ECT) genes by 1231A3 iPSCs, induced NCCs on days 2–10 (D2, D4, D6, D8, D10), and the high (H) and low (L) fractions of CD271-positive cells. **B** A heatmap illustrating the expression of mesoderm (MES) and endoderm (END) genes for 1231A3 iPSCs, induced NCCs on days 2–10 (D2, D4, D6, D8, D10), and the high (H) and low (L) fractions of CD271-positive cells. **C** A heatmap illustrating the expression of regional markers, forebrain (FB), midbrain (MB), midbrain hindbrain border (MHB), hindbrain (HB), and spinal cord (SC) genes for 1231A3 iPSCs, induced NCCs on days 2–10 (D2, D4, D6, D8, D10), and high (H) and low (L) fractions of CD271-positive cells. **D** A PCA analysis of NCC induction from 1231A3 iPSCs. PC1: principal component 1, PC2: principal component 2. All sequence data were available in GEO (GSE206048).

To investigate the developmental pathway followed by induced NCCs, the global gene expression profiles of NCCs on days 2, 4, 6, 8, and 10 post induction and those of CD271^high^ and CD271^low^ cells on day 10 were analyzed. During the first 6 days, the expression of pluripotent markers, such as *POU5F1*, *NANOG*, *ZFP42*, *DNMT3B*, and *CDH1*, was downregulated (Fig. 2A). Conversely, the expression of ectodermal markers, including neuroectoderm (*PAX6* and *DACH1*), neural plate border (*PAX3*, *PAX7*, *ZIC1*, *MSX2*, and *TFAP2A*), and NCC (*SOX10*, *FOXD3*, *NGFR*, *ITGA4*, and *SNAI2*) markers, was upregulated. Consistent with our RT-qPCR data, CD271^high^ cells highly expressed NCC markers, whereas CD271^low^ cells highly expressed neuroectodermal markers. These data confirm the successful enrichment of NCCs by CD271 FACS. The activation of an epidermal ectoderm marker (*ECT*), mesoderm markers (*T*, *MIXL1*, *TBX6*, *WNT3*, *SIM1*, *OSR1*, and *KDR*), and endoderm markers (*FOXA2*, *SOX17*, *CER1*, and *LHX1*) was marginal (Fig. 2B). The expression of region-specific homeobox genes revealed that our protocol induced cells in the midbrain and anterior hindbrain regions (positive for *OTX1*, *OTX2*, *EN1*, and *HOXA2*) but not in the forebrain or spinal cord (Fig. 2C). A principal component analysis (PCA) showed that a shift along primary component 1 (PC1, 39.2%) was observed during the first 6 days, whereas a major shift was observed along with PC2 (16.1%) from day 6 to day 10 (Fig. 2D). Taken together, these data suggest the directed differentiation of the midbrain and anterior hindbrain NCCs through the ectodermal lineage and dynamic fate restriction from pluripotency to neuroectoderm and NCCs during the first 6 days.

### Xeno-free NCCs expanded with the TGFβ inhibitor SB431542, EGF, and bFGF lost their neural differentiation capacity

**Fig. 3:**
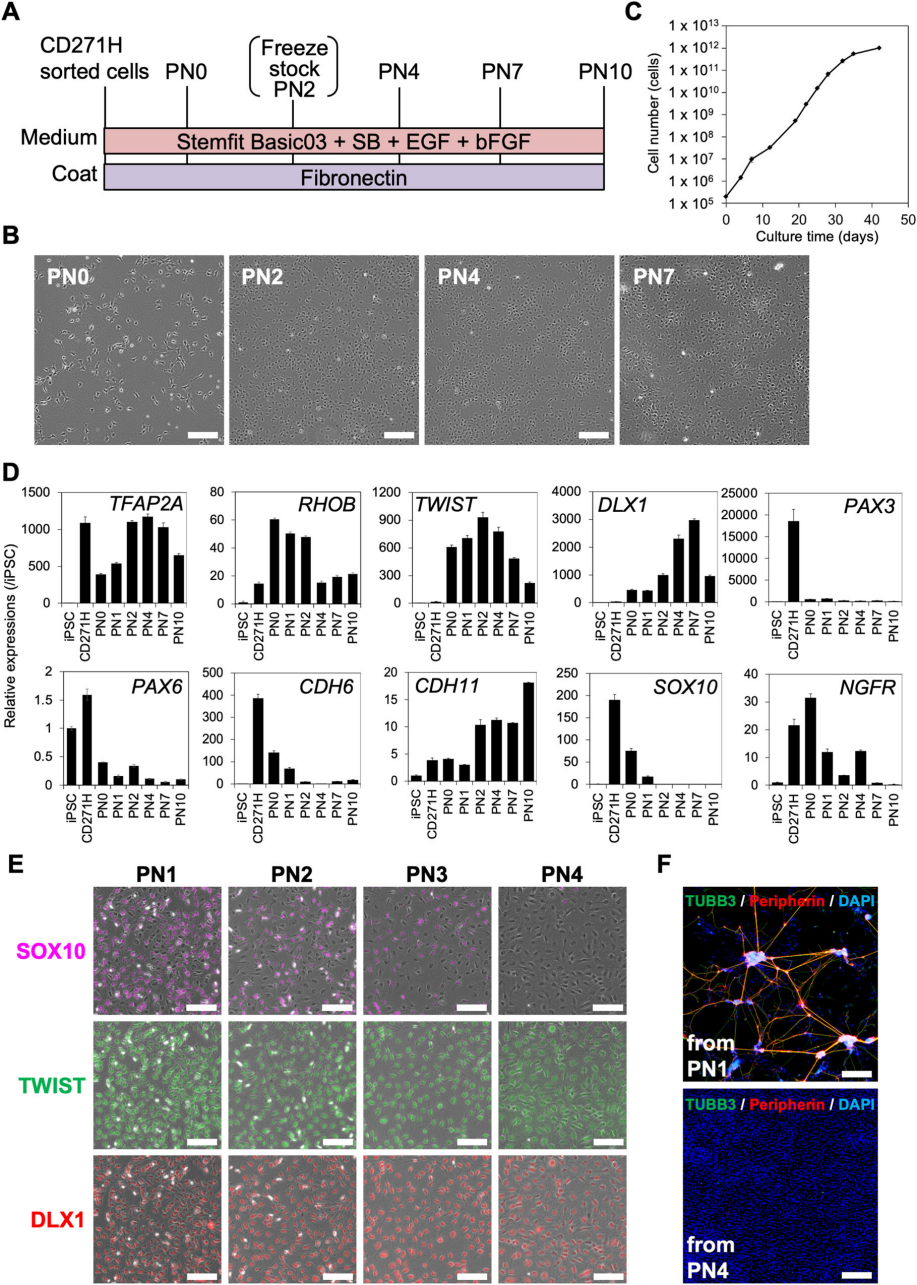
Xeno-free conditions to expand iNCCs that have lost neural differentiation potency. **A** Schematic representation of the neural crest expansion culture protocol. **B** Phase contrast images of passage numbers (PN) 0, 2, 4, and 7. Scale bars, 100 µm. **C** Number of cells during the expansion culture. Data are the mean ± SD, *n* = 3. **D** The expression of marker genes during the expansion culture. The mRNA expression of each gene was analyzed by RT-qPCR during the expansion culture and is shown relative to the expression level in 1231A3 iPSCs. Data are the mean ± SD, *n* = 3. **E** Immunostaining of SOX10 (purple) TWIST (green) and DLX1 (red) during the expansion culture. Scale bar, 100 µm. **F** Peripheral neuron differentiation from PN1 (upper panel) or PN4 (lower panel) of the expansion culture. Cells were stained with anti-TUBB3 antibody (green) and anti-Peripherin antibody (red). Nuclei were stained with DAPI (blue). Scale bar, 200 µm.

Previous reports have shown that induced NCCs can be expanded in a chemically defined medium (containing BSA) with the TGFβ inhibitor SB, EGF, and bFGF on fibronectin-coated dishes^9,21^. Here, we investigated whether a xeno-free basal medium—Basic03—supplemented with SB, EGF, and bFGF could also expand NCCs (Fig. 3A). Under these conditions, cells proliferated and maintained a similar fibroblastic morphology for several passages (usually until around passage 7) (Fig. 3B, C). However, the proliferative speed decreased and eventually ceased.

To check whether expanded cells maintained their NCC characteristics, the expression of NCC markers was analyzed (Fig. 3D). *TFAP2A*, a pan-NCC marker, was expressed in CD271^high^ cells and maintained until passage 10, suggesting that NCC characteristics were, at least in part, maintained during passages. The expression of *RHOB*, another NCC marker, was observed in CD271^high^ cells, peaked in early passages, and maintained until passage 10, also suggesting that NCC characteristics were maintained. The expression levels of *TWIST* and *DLX1*, markers of migrating NCCs, were low in CD271^high^ cells but expressed from passage 0 to passage 10, although they peaked at passage 2 and passage 7, respectively. *PAX3*, a marker of pre-migratory and early migrating NCCs, was expressed just after sorting, but its level significantly decreased after plating. The expression of *PAX6*, a marker of neuroectoderm, was low and comparative to that in iPSCs. These data suggest that the characteristics of NCCs changed gradually from pre-migratory to migratory during passage. In accordance with this idea, the switch of cadherin from *CDH6* to *CDH11* was observed with passaging. Strikingly, however, the expression of *SOX10*, a neural crest stem cell marker^22^, was significantly downregulated during early passages. *NGFR*, a pre-migratory and migratory NCC marker, was highly expressed in the early passages but hardly detected at passage 7. These data were also confirmed by a transcriptome analysis of markers for PSCs, pre-migratory NCCs, post-migratory NCCs, pan-NCCs and EMT (Supplementary Fig. 3A) and immunocytochemistry with anti-SOX10, anti-TWIST, and anti-DLX1 antibodies (Fig. 3E). All data support the idea that cell characteristics change gradually with passaging.

DLX1 and CDH11 are markers for mesenchymal cells, and, since their expression increased at later passages, we hypothesized that expanded NCCs gained mesenchymal characteristics. To test this hypothesis, the differentiation properties of expanded NCCs were assessed by culturing NCCs under neuron, glia, melanocyte and MSC induction conditions. As expected, NCCs differentiated to form peripheral neurons and glia at passage 1 but not at passage 4 (Fig. 3F and Supplementary Fig. 3B). Melanocytes were not differentiated from NCCs at passages 1 or 4 (Supplementary Fig. 3B). These results suggested that NCCs cultured in our expansion culture first lose their ability to differentiate into pigment cells and then into peripheral neurons and glia.

### Differentiation of MSCs from expanded xeno-free NCCs

**Fig. 4:**
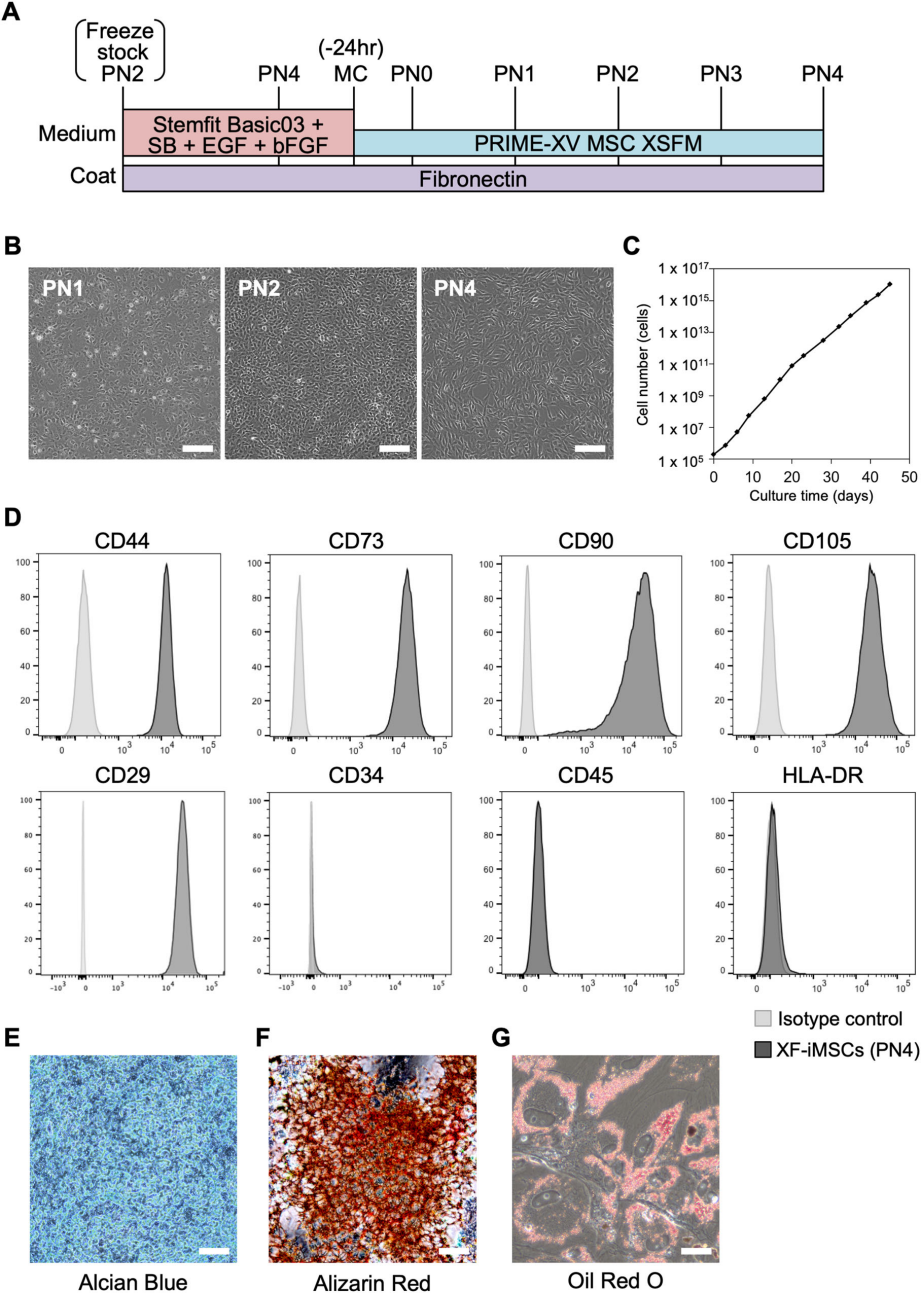
Xeno-free induction of iMSCs from expanded iNCCs. **A** Schematic representation of the MSC induction protocol using PRIME-XV MSC XSFM xeno-free medium. **B** Phase contrast images of passage number (PN) 1, 2, and 4. Scale bar, 100 µm. **C** Number of cells during the MSC induction. Data are the mean ± SD *n* = 3. **D** The expression of hMSC-related surface markers in XF-iMSCs (dark gray) and isotype control (gray) at PN4. **E**–**G** Differentiation properties of XF-iMSCs. The induction of chondrogenic, osteogenic, and adipogenic lineages was evaluated by alcian blue staining (**E**), alizarin red staining (**F**), and oil red O staining (**G**), respectively. Scale bar, 50 µm.

When we cultured NCCs with serum-containing medium or xeno-free MSC medium (PRIME-XV® MSC Expansion XSFM) (Fig. 4A), NCCs at passages 2, 4 and 7 proliferated exponentially (Fig. 4B, C, and data not shown). They also showed a morphology (Supplementary Fig. 4A) and gene expression profile consistent with MSCs (Supplementary Fig. 4B, C). However, compared to cells derived from NCCs at passage 4, a significant number of cells died within a few days when induced from NCCs at passage 2 (Supplementary Fig. 4D). Therefore, for further characterization, we used cells from NCCs at passage 4. We also checked for MSC surface markers and confirmed that cells derived from NCCs at passage 4 were positive for CD44, CD73, CD90, CD105, and CD29 and negative for CD34, CD45 and HLA-DR at passage 4 (Fig. 4D). Those cells derived from xeno-free medium (XF-iMSCs, hereafter) at passage 4 could differentiate into cartilage, bone, and adipose under chondrogenic, osteogenic, and adipogenic induction conditions, respectively (Fig. 4E–G). These data suggest that the expansion of NCCs restricts their fate to mesenchymal lineages.

### Comparison of XF-iMSCs and tissue-derived MSCs

MSCs isolated from different tissues or prepared by different methods have different characteristics. Therefore, we compared our XF-iMSCs with various types of MSCs. First, we performed a transcriptome analysis of XF-iMSCs and tissue-derived primary human MSCs (bone marrow-derived MSCs (hBM-MSCs), adipose-derived MSCs (hAC-MSCs), and umbilical cord-derived MSCs (hUC-MSCs)) (Supplementary Fig. 5A), finding that although only XF-iMSCs expressed neural progenitor genes and neural lineage genes, there were substantial similarities in the MSC marker expression and global gene expression profiles (Supplementary Figs. 5B–D and 6). Next, we added IFN-γ to these MSCs and performed a qPCR for the immunomodulators IDO1 and PD-LI and ELISA for PGE2 (Supplementary Fig. 7) to check the response to proinflammatory cell factors. Although the cell morphology remained almost unchanged (Supplementary Fig. 7A), the expression of *IDO1* and *PD-LI* was upregulated by IFN-γ treatment in all examined MSC types (Supplementary Fig. 7B). Interestingly, PGE2 secretion was increased in XF-iMSCs and UC-MSCs but decreased in BM-MSCs and AC-MSCs (Supplementary Fig. 7C), suggesting that XF-iMSCs are closer to the UC-MSCs.

### Contribution of XF-iMSCs to skull bone regeneration

**Fig. 5:**
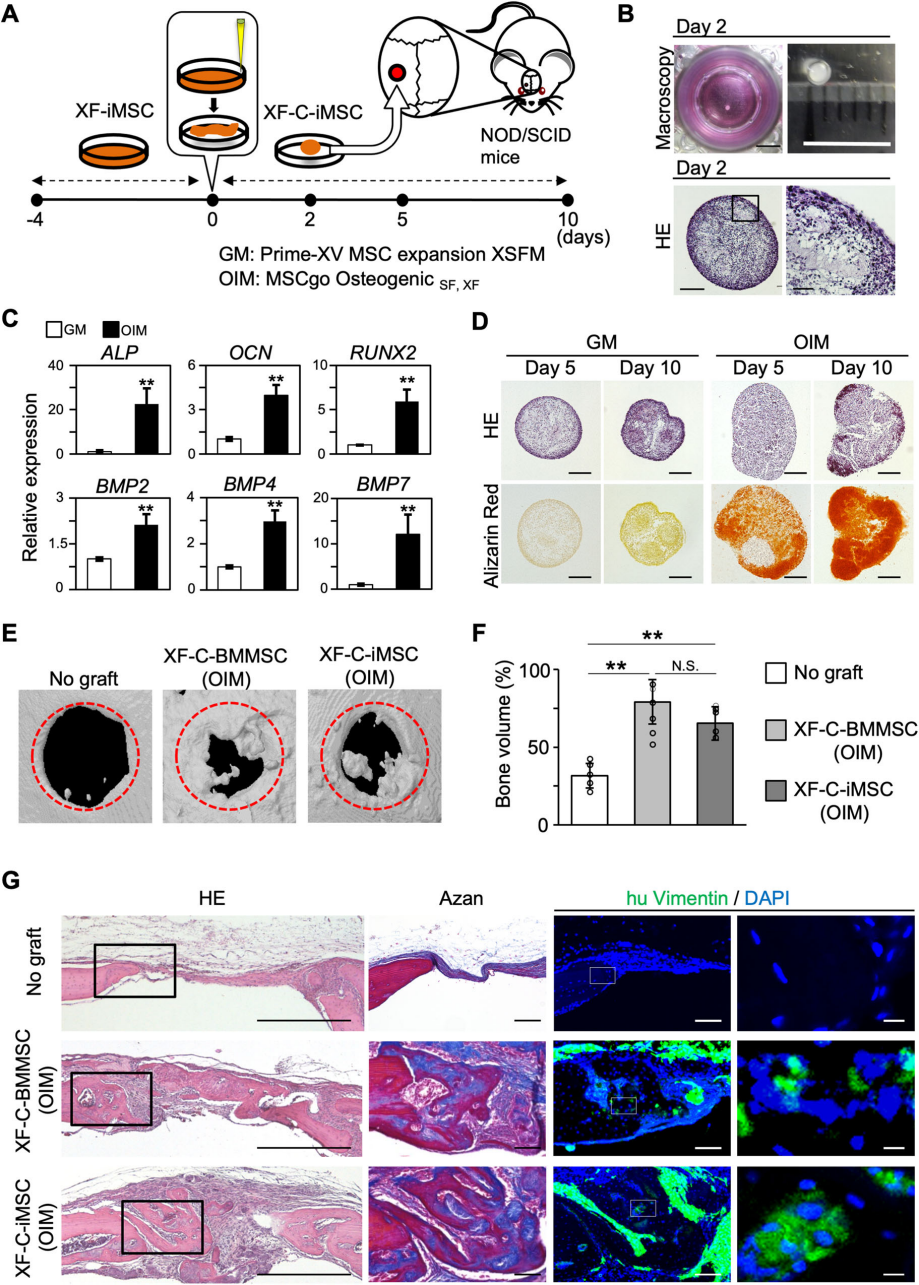
Enhanced skull bone regeneration by transplanted osteogenic clump-XF-iMSCs. **A** Schematic representation of the transplantation of XF-iMSCs into mouse skulls. **B** Shapes (upper panel) and sectional images of XF-C-iMSCs stained by HE (lower panel). Scale bars, 5 mm (upper left and right), 100 µm (lower left), and 10 µm (lower right). **C** The expression marker genes in GM cultured clumps (white) and OIM cultured clumps (black). The mRNA expression of each gene was analyzed by RT-qPCR in GM cultured clumps and OIM cultured clumps and is shown relative to the expression level in GM cultured clumps. Data are the mean ± SD, *n* = 4. **P < 0.01. **D** Sectional images of GM and OIM cultured clumps stained by HE (upper panel) or alizarin red (lower panel). Scale bars, 200 µm. **E** CT-scanning images of transplanted mouse skulls. Trepanned areas are indicated by the red circles. **F** Relative bone volume after no graft (white), XF-C-BMMSC transplantation (gray), and XF-C-iMSC transplantation (black). Data are the mean ± SD, n = 6. **P < 0.01. n.s. not significant. **G** Lateral section images of the transplanted skull. Sections were stained by HE (left column), Azan (middle column) or anti-human vimentin (right column). Nuclei were stained with DAPI (blue). Black boxes in the left column show the same areas as those in the middle column in the serial sections. The right column shows high magnification images of the white box in the middle column. Scale bars, 500 µm (left column), 100 µm (middle column), and 10 µm (right column).

To investigate the function of XF-iMSCs, their in vitro differentiation potential and in vivo regeneration ability for bone was examined. XF-iMSCs were cultured in a 48-well tissue culture plate with xeno-free MSC medium for 4 days, manually detached from wells as sheets, and cultured for 2 days in xeno-free MSC medium (GM) or osteocyte induction medium (OIM) to allow for the formation of clumps (XF-Clump-iMSCs; XF-C-iMSCs, hereafter) (Fig. 5A, B)^23^. On day 2, the clumps were 1 mm in diameter and contained a rich extracellular matrix (Fig. 5B). After being cultured in xeno-free osteogenic medium for three more days, XF-C-iMSCs expressed osteogenic markers (*ALP*, *OCN*, *RUNX2*, *BMP2*, *BMP4*, and *BMP7*) (Fig. 5C). In vitro osteogenic differentiation was confirmed by alizarin red staining on day 5 (osteogenic induction for three days) and day 10 (osteogenic induction for 8 days) (OIM in Fig. 5D). To assess the bone regeneration ability, XF-C-iMSCs (OIM) at 5 days were transplanted into a 1.6-mm hole of the skull bone of immunodeficient non-obese diabetic/severe combined immunodeficiency (NOD/SCID) mice. This time, clumps of hBM-MSCs maintained in xeno-free MSC medium and induced to osteogenesis (XF-C-BMMSCs (OIM)) were used as a positive control. Four weeks after the transplantation, mineralized areas were recovered by the XF-C-iMSCs (OIM) transplantation, similar to XF-C-BMMSCs (Fig. 5E, F, Supplementary Fig. 8). Although histological and immunohistochemical analyses revealed the contribution of iMSCs and hBM-MSCs in regenerated bone regions, the majority of regenerated bone was negative for anti-human-specific vimentin in both cases (Fig. 5G). Similar results were observed when XF-C-iMSCs cultured with MSC medium were transplanted (Supplementary Fig. 9). These results suggest that, although XF-iMSCs could differentiate into bone in vivo, the main therapeutic effect was a paracrine effect in this skull model.

### Enhanced skeletal muscle regeneration by XF-iMSC transplantation

**Fig. 6:**
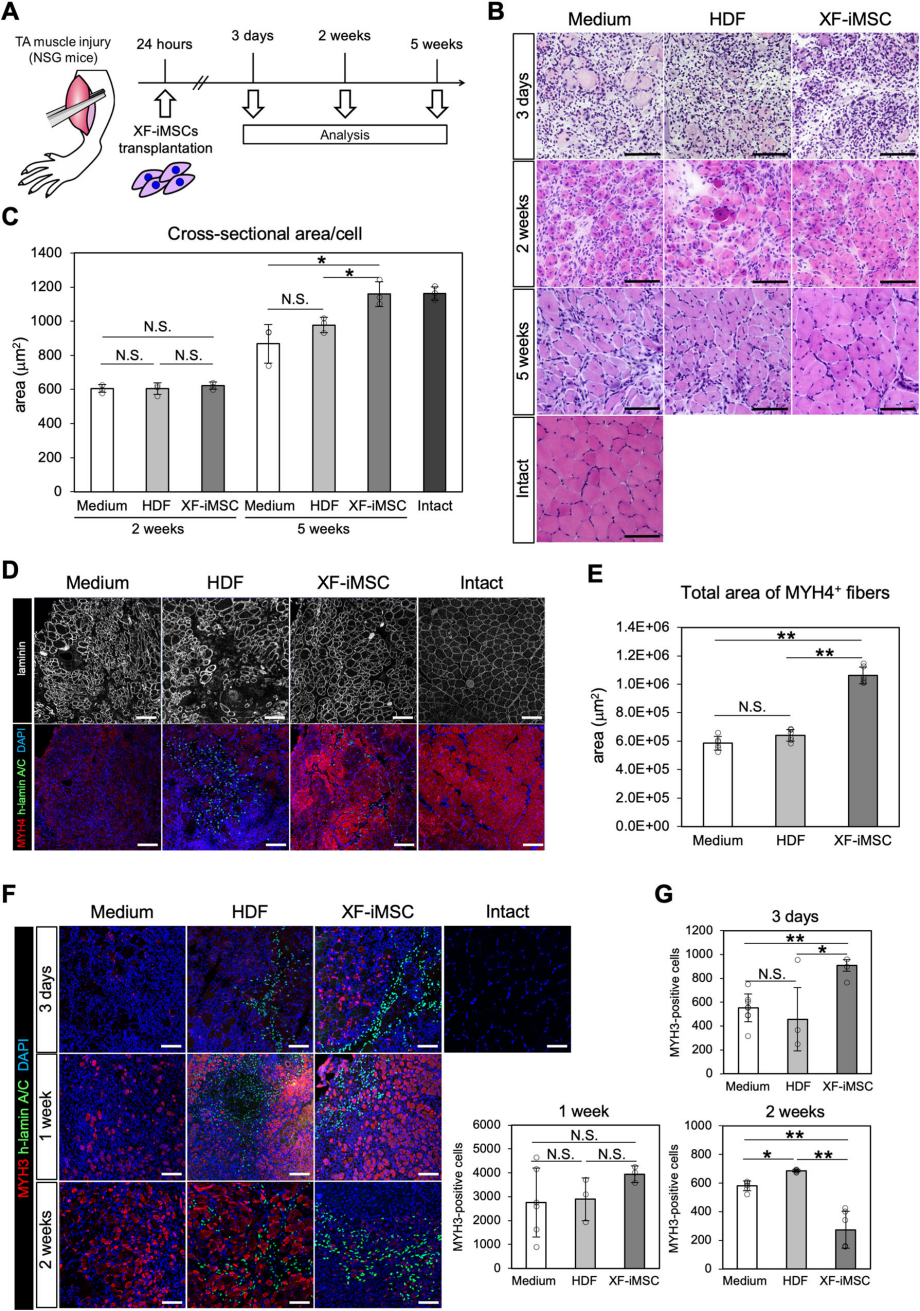
Early reactivation of myogenic markers during skeletal muscle regeneration after XF-iMSCs transplantation. **A** Schematic representation of the transplantation of XF-iMSCs into injured skeletal muscle [Tibialis anterior (TA) muscle]. **B** Sectional images of injured TA muscle at 3 days, 2 weeks, and 5 weeks after medium (left column), HDF (middle column), and XF-iMSC (right column) transplantation. Intact: uninjured NSG mouse. Sections were stained by HE. Scale bars, 100 µm. **C** Cross-sectional area per cell in the injured field 2 or 5 weeks after transplantation. Data are the mean ± SD, *n* = 3. *P < 0.05. n.s. not significant. **D** Sectional images of the injured TA muscle 2 weeks after transplantation. Sections were stained with anti-laminin-α2 antibody (white), anti-MYH4 antibody (red), and anti-human LaminA/C antibody (green). Nuclei were stained with DAPI (blue). Scale bars, 100 µm. **E** Total area of MYH4-positive fibers 2 weeks after transplantation. Data are the mean ± SD, *n* = 6. **P < 0.01. n.s. not significant. **F** Sectional images of injured TA muscle at 3 days, 1 week, and 2 weeks after transplantation. Sections were stained with anti-MYH3 antibody (red) and anti-human LaminA/C antibody (green). Nuclei were stained with DAPI (blue). **G** Numbers of MYH3-positive cells at 3 days, 1 week, and 2 weeks after transplantation. Data are the mean ± SD *n* = 3 (3 days), *n* = 3 (1 week), *n* = 6 (2 weeks). *P < 0.05; **P < 0.01; n.s. not significant.

To assess the impact of iMSCs on the regeneration of other tissues, XF-iMSCs were transplanted into the tibialis anterior (TA) muscle crush model of immunodeficient NOD/SCID/IL2Rgamma null (NSG) mice (Fig. 6A). After 24 h, XF-iMSCs and human dermal fibroblasts (HDFs) were transplanted into TA-injured mice. Three days after injury (2 days after transplantation), muscle fibers were degenerated at the injury site, and we observed no major histological differences between the control groups (medium injection and HDF transplantation groups) and the XF-iMSC transplantation group (Fig. 6B). After 2 weeks, there were also no differences among the groups in terms of histology and the average cross-sectional area of muscle fibers (Fig. 6B, C). At 5 weeks, however, the average cross-sectional area of muscle fibers was significantly larger in the XF-iMSC transplantation group than those in control groups and was comparable to that of intact muscle fibers, although nuclei were found in the center of each muscle fiber. These results suggest that XF-iMSC transplantation encourages accelerated skeletal muscle regeneration.

For the molecular characterization of the regeneration processes, immunohistochemical analyses were performed with anti-MYH4 (a mature muscle fiber marker) and anti-MYH3 (an embryonic and fetal muscle fiber marker, also expressed in regenerating muscle fibers) antibodies as well as anti-laminin antibody (basement membrane marker). At 5 weeks, muscle fibers with large diameters were stained with MYH4, which is consistent with MYH4 being a marker for mature skeletal muscle (Supplementary Fig. 10A). No staining was detected with MYH3 antibody, which is consistent with MYH3 being a marker expressed in the early phase of muscle regeneration (Supplementary Fig. 10B). At 2 weeks, although anti-laminin staining revealed that the cross-sectional area of skeletal muscle cells in the XF-iMSC-transplanted group was smaller than that in intact skeletal muscle, the number of MYH4-positive cells was increased compared with the control groups (Fig. 6D, E). The nuclei of MYH4-positive cells did not co-stain with human-specific lamin A/C (h-lamin A/C), which marks the nucleus of transplanted human cells, suggesting that transplanted XF-iMSCs did not differentiate into skeletal muscle cells. During the early stages, on day 3, MYH3-positive cells were increased in the XF-iMSC-transplanted group compared with those in the control groups (Fig. 6F, G). After 1 week, the number of MYH3-positive cells was higher in the XF-iMSC transplantation group than in the control group. In contrast, the number of MYH3-positive cells was lower in the XF-iMSC transplantation group than in the control group after 2 weeks. These data suggest that XF-iMSC transplantation promotes early recovery from muscle damage. The nuclei of MYH3-positive cells was never co-stained with h-lamin A/C; however, we noticed that the majority of MYH3-positive cells were tended to localize near the h-lamin A/C-positive cells at day 3 (Supplementary Fig. 11). These data suggest that the contribution of XF-iMSCs to muscle regeneration occurs via paracrine factors.

### Accelerated myotube differentiation by XF-iMSC-conditioned medium in vitro

**Fig. 7:**
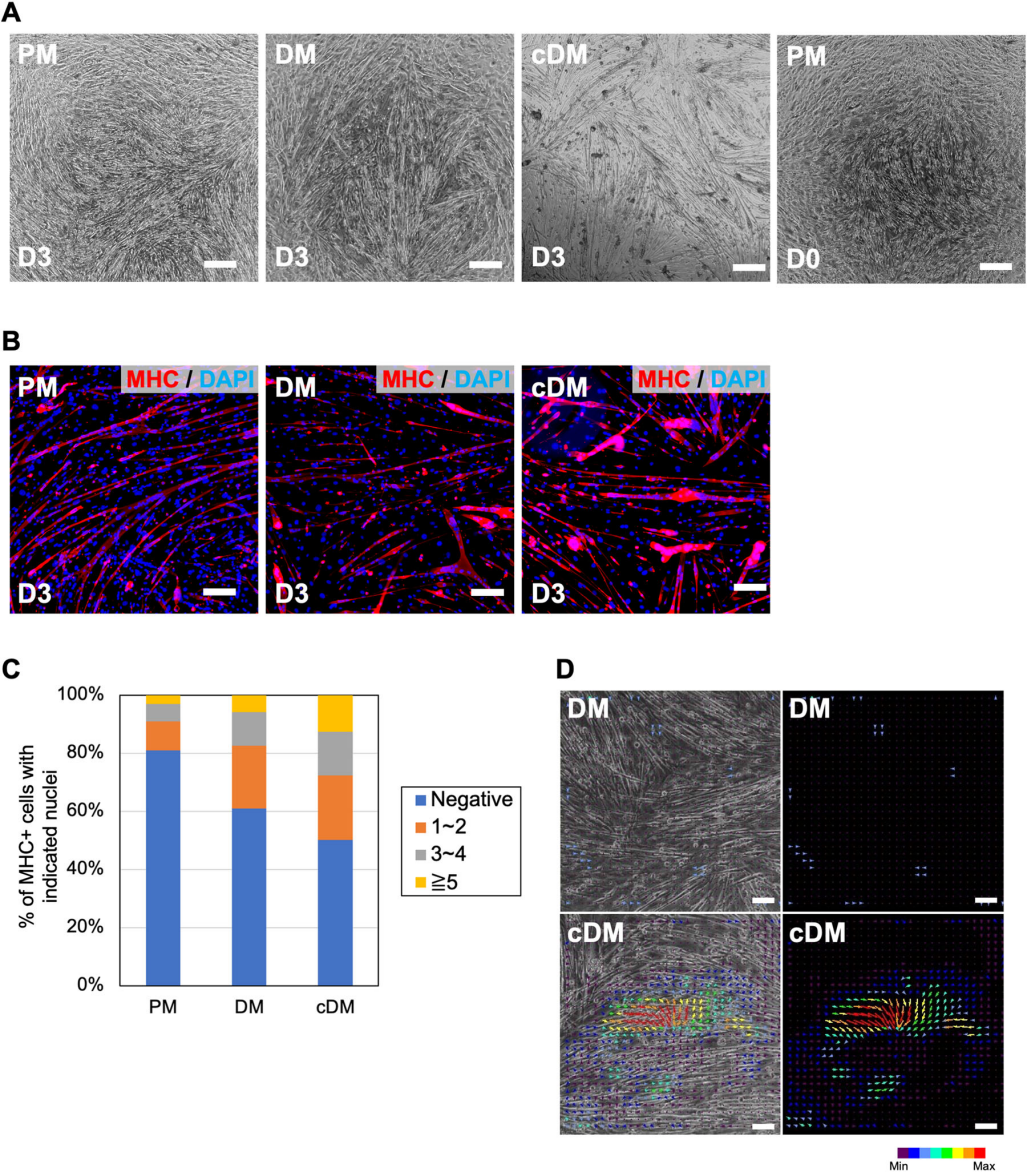
Effect of XF-iMSC-conditioned medium on myotube differentiation of newborn mouse myoblasts. **A** Phase contrast images of myoblasts (PM: Proliferation Medium) cultured with DM (DM Differentiation Medium) or cDM (cDM: XF-iMSC conditioned differentiation Medium) for 3 days. Scale bar, 100 µm. **B** Immunostaining images of mouse myoblast cultured with DM or cDM. Cells were stained with anti-MHC antibody (red). Nuclei were stained with DAPI (blue). Scale bars, 100 µm. **C** Percentage of MHC-positive cells with indicated nuclei. Negative (Blue) means MHC-negative cells. **D** Phase contrast images (left panel) and movement calculated by ImageJ (right panel) of myoblasts cultured in DM or cDM for 3 days.

**Fig. 8:**
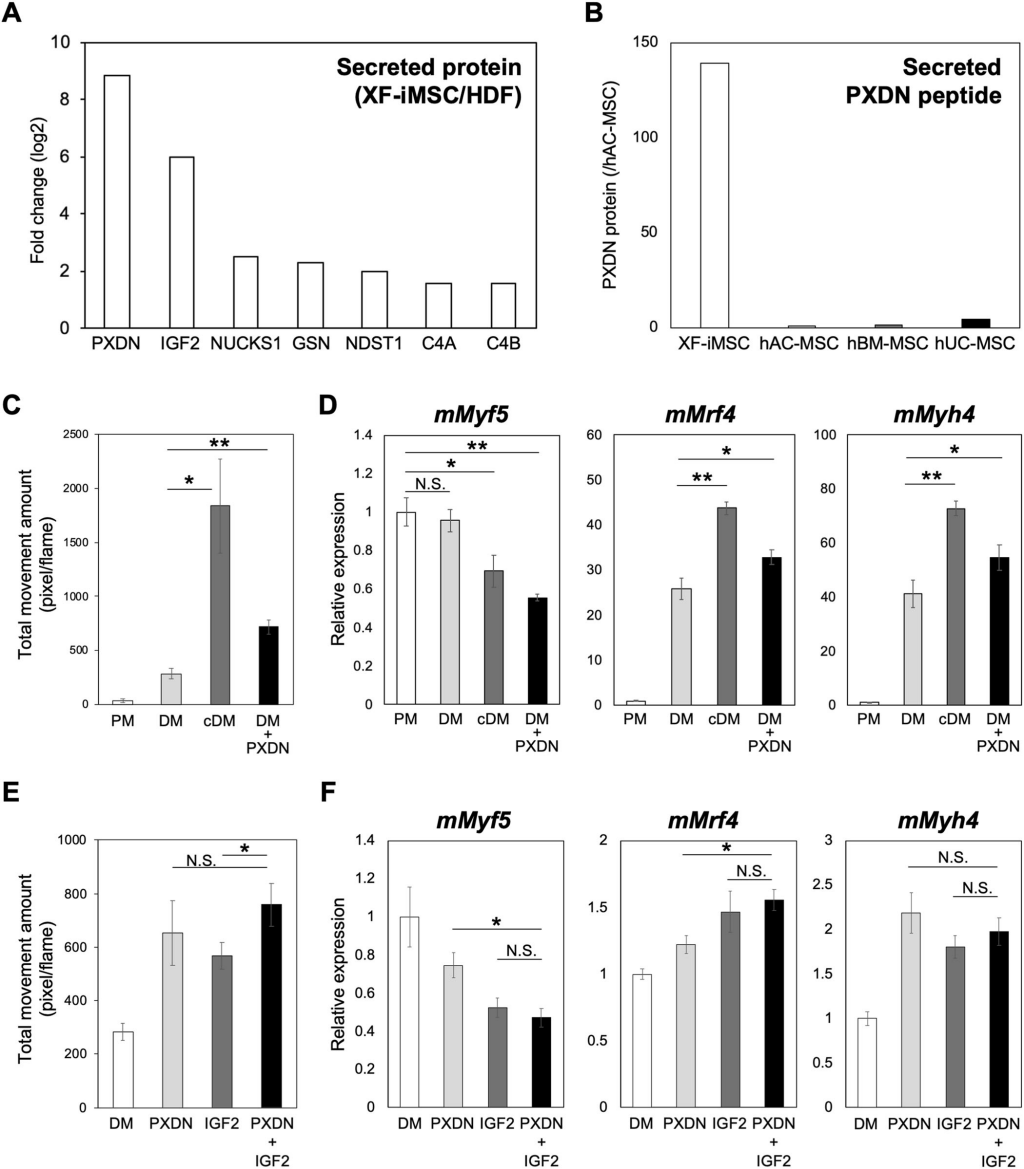
Effect of XF-iMSC-secreted molecules on myotube differentiation of newborn mouse myoblasts. **A** Difference in the amount of secreted proteins between XF-iMSC- and HDF-conditioned media. **B** Amount of secreted PXDN peptide in XF-iMSC, hAC-MSC, hBM-MSC, and hUC-MSC cultured media. Data are shown relative to the expression level in hAC-MSC. Proteome data were available in jPOSTrepo (JPST001693). **C** Total cell movement of myoblasts cultured in PM (Proliferation Medium), DM (Differentiation Medium), cDM (XF-iMSC conditioned Differentiation Medium), and DM + PXDN (0.5 µM) for 3 days. Data are the mean ± SD, *n* = 4. *P < 0.05; **P < 0.01. **D** The expression of marker genes during myotube differentiation. The mRNA expression of each gene was analyzed using RT-qPCR during myotube differentiation and shown as the relative expression level in PM. Data are the mean ± SD, *n* = 3. *P < 0.05; **P < 0.01; n.s. not significant. **E** Total movement of myoblasts cultured in DM, PXDN, IGF2, and PXDN + IGF2 for 3 days. Data are the mean ± SD, *n* = 4. *P < 0.05; n.s. not significant. **F** Expression of marker genes during myotube differentiation. The mRNA expression of each gene was analyzed by RT-qPCR during myotube differentiation and is shown relative to the expression level in DM. Data are the mean ± SD, *n* = 3. *P < 0.05; n.s. not significant.

To check whether soluble factors secreted by XF-iMSCs regulate the early activation of MYH3 in vivo, we injected XF-iMSC-conditioned medium into injured TA muscle. Although a subtle increase was observed in the number of MYH3-positive cells (Supplementary Fig. 12), there was no significant difference between intact and conditioned media. Since in vivo the conditioned media could have diffused, degraded, or been of low concentration when injected into mouse TA muscle, we employed an in vitro differentiation system to assess the paracrine effects of XF-iMSCs on muscle regeneration. Primary murine myoblasts were isolated from neonates and cultured with proliferation medium (PM), differentiation medium (DM), and XF-iMSC-conditioned DM (cDM). Cells cultured with DM or cDM formed myotube-like structures, whereas cells cultured with PM did not (Fig. 7A–C). Intriguingly, cells cultured with cDM showed contraction more frequently than those cultured with DM after treatment with fresh medium (Fig. 7D and Supplementary Movie 1–3). To reveal the molecular nature of cDM, a proteome analysis was performed using the supernatants of XF-iMSCs and HDFs, and proteins whose secretion was increased by XF-iMSCs were identified (peroxidasin (PXDN), IGF2, NUCKS1, GSN, NDST1, C4A, and C4B) (Fig. 8A). Among these proteins, we focused on PXDN and IGF2 because (1) they had the largest difference in the amounts of secretion from XF-iMSCs and HDFs and (2) they had a significantly higher secretion from XF-iMSCs compared with adult human MSCs (Fig. 8B and Supplementary Fig. 6). Cells cultured with DM supplemented with PXDN protein also showed more frequent contractions than those cultured with DM alone (Fig. 8C and Supplementary Movie 4). Consistent with these observations, RT-qPCR revealed less expression of a myoblast marker (*mMyf5*) and more expression of myotube markers (*mMrf4* and *mMyh4*) (Fig. 8D). We also conducted a single administration of IGF2 and a dual administration of PXDN and IGF2 and observed muscle contraction and the expression of myoblast markers (Fig. 8E, F). We found that IGF2 alone was as effective as PXDN, but the effect was not as strong as cDM, and no synergistic effect was observed with the dual administration. These data indicate that myotubular differentiation from myoblasts is accelerated by proteins secreted from XF-iMSCs and that PXDN and IGF2 play roles in this, at least in part.

## Discussion

Here, we report the successful production of NCC-derived MSCs from iPSCs under xeno-free conditions. In fact, there have been many papers describing iPSC-derived MSCs^24–27^, and such MSCs have already been used in human^28^. However, our protocol is original in terms of the staged differentiation through NCCs. Although the protocol remains to be improved in the future, considering the time-consuming sorting procedure and the costly fibronectin coating, this method has a theoretical advantage compared to the ordinary method in the ability of cryopreservation and quality check at this intermediate stage, and of CD271^high^ sort enriching CD271-positive cells at >99% purity (Supplementary Fig. 1E).

Although we showed the robustness of our protocol using several iPSC lines, the induction efficiency varied (Supplementary Fig. 1D). There are at least three explanations for this effect. First, human iPSCs have clonal variations associated with epigenetic status^29^, which would be reflected in the induction efficiency of NCCs. Second, fine regulation of the BMP signal levels is important for NCC induction^30^; modulating the BMP signal intensity may improve induction efficiency. Third and finally, the seeding cell number and preculture periods were not optimized. Interestingly, iPSC clones with low CD271^high^ induction efficiency tended to have a large CD271^low^ population (data not shown). Since this population contains neuroectoderm (Figs. 1E and 2A), the poor induction efficiency of CD271^high^ might correlate to and be explained by the induction balance between neuroectoderm and NCCs.

XF-iMSCs have the potential to accelerate damaged bone and skeletal muscle repair. The majority of regenerated bone and regenerated skeletal muscle were derived from host cells, and regeneration occurred near the transplanted cells. We identified PXDN and IGF2 as secreted proteins from XF-iMSCs that partially contribute to myotube differentiation. Taken together, our findings indicate that XF-iMSCs with paracrine effects are useful for the future application of cell transplantation medicine.

In this study, we found a gradual change in gene expression during NCC expansion culture (Fig. 3D, E). *DLX1*, *TWIST*, and *CDH11* are markers for late migrating NCCs found in the branchial arches, suggesting a transition of characteristics from neural crest stem cells to mesenchymal fate-restricted NCCs. Similar expressional changes were found in BSA-containing medium when previous data (GSE 60313) was reanalyzed, suggesting that the change is not specific for xeno-free medium. We found that the differentiation capacity of neurons was lost in passaged NCCs, which supports this conclusion (Fig. 3F). We hypothesize the existence of a pro-MSC state, wherein cells express both migratory NCC markers (*TFAP2A, TWIST, DLX1*) and a mesenchymal marker (*CDH11*). We believe that expanded NCCs are in the pro-MSC state (Fig. 3D).

Since NCCs are present only during embryonic development, their functions in morphogenesis during the developmental stage have been studied; however, their functions at later stages have not been studied. Recently, however, several studies have shown that NCC lineage cells play important roles in vertebrate tissue repair. In the process of regenerating mandibular bone, skeletal stem cells have been shown to reactivate NCC-like characteristics by sensing mechanotransduction via focal adhesion kinase signaling and promoting regeneration of the jaw^31^. In addition, studies have shown that mesenchymal cells derived from NCCs in vivo exist in the peripheral nervous system and contribute to bone and skin regeneration by migrating to the relevant tissues in response to tissue damage^32^. Since the peripheral nervous system is distributed throughout the body, NCC-derived stromal cells in the endoneurium may be involved in various tissue regeneration activities beyond bone and skin regeneration^33^. In this study, we have shown the potential contribution of NCC-derived MSCs for skull bone regeneration and skeletal muscle regeneration. Taken together with the findings of the abovementioned reports, our findings support our pro-MSC hypothesis.

Regarding the in vivo regenerative function of XF-iMSCs, we were interested in identifying the paracrine factors involved in tissue regeneration. In skull bone regeneration, XF-C-iMSCs cultured with osteogenic induction medium expressed BMP2, BMP4, and BMP7 (Fig. 5C). Since BMP was originally identified as a bone inducer^34,35^, BMPs secreted from XF-C-iMSCs (OIM) could contribute to skull bone regeneration. However, we also found that XF-C-iMSCs cultured with MSC medium were able to regenerate skull bone (Supplementary Fig. 9), even though the regenerated bone was thinner than that in the XF-C-iMSC (OIM) group. Taken together these findings indicate that factors other than BMPs might also be involved in stimulating bone regeneration. Further research is required to elucidate the mechanisms involved in this process.

In muscle regeneration, there are two possible mode-of-actions for how XF-iMSCs promote the recovery of mouse muscle: promotion of muscle differentiation, and protection against inflammatory damage. Regarding the latter, our previous published work showed that iMSCs have an immunosuppressive effect both in vivo and in vitro^36,37^, although the molecular nature is unknown. Regarding the former, in the normal regenerative processes, quiescent muscle stem cells (satellite cells) become active following proliferation, differentiation, and fusion^38^. These processes are regulated by several cytokines secreted by the surrounding cells. In vitro co-culture studies have shown that endothelial cells may be a source of insulin growth factor 1, vascular endothelial growth factor, hepatocyte growth factor, platelet-derived growth factor, and fibroblast growth factor and that they may promote satellite cell growth. Macrophages secrete tumor necrosis factor α, interferon γ, and interleukin (IL) 6 to promote myoblast proliferation. IL4 and IL10, secreted from macrophages that have phenotypically switched to an anti-inflammatory fate also play a role in muscle regeneration by stimulating the differentiation and fusion of myoblasts. Bipotent mesenchymal fibroadipogenic progenitors (FAPs), which are characterized by PDGFRα and CD34 expression and located in the muscle interstitium, are also important for muscle regeneration^39–41^. FAPs secrete WNT1-inducible signaling pathway protein (WISP1), a matricellular protein that controls muscle stem cell amplification and commitment^42^. Intriguingly, XF-iMSCs express *PDGFRα*, *CD34*, and *WISP1* (Supplementary Fig. 13), which may contribute to muscle regeneration in the same way as FAPs. The developmental origin of FAPs is still unclear, but one fascinating hypothesis is the neural crest origin theory.

In this study, we identified PXDN and IGF2 as protein secreted from XF-iMSCs that stimulate in vitro myoblast differentiation, at least partially (Fig. 8). PXDN is strongly expressed in skeletal muscle and heart^43^ and regulates sulfilimine crosslinks and dense fibrillar network assembly of not only Col IV but also fibronectin and laminin^44^. However, there is a PXDN KO mouse that has an ophthalmic phenotype but nothing else suggestive of a role in muscle developmental biology or regeneration or for any other tissue for that matter. Moreover, the power of DM supplemented with PXDN, IGF2 or both was limited in terms of the differentiation and muscle contraction, suggesting neither are critical factors. More experiments are needed to fully define the molecular mechanisms for muscle regeneration stimulated by XF-iMSCs.

## Methods

### Human iPSCs and human primary cultured cell lines

Human iPSCs (1231A3, 1381A5, 1381B5, 1383D2, and 1383D10, reprogrammed with episomal vectors, kindly provided from Yamanaka laboratory) were cultured on an iMatrix-511 (Nippi, Tokyo, Japan)-coated cell culture plate or dish in StemFit AK03N (Ajinomoto, Tokyo, Japan), as described previously^20^. The medium was changed every 2 days. Human mesenchymal stem/stromal cells (hAC-MSCs, hBM-MSCs, hUC-MSCs) were obtained from PromoCell (Heidelberg, Germany). MSCs were cultured on fibronectin (Millipore, Bedford, CA, USA)-coated culture dishes in PRIME-XV MSC Expansion XSFM medium (FUJIFILM Irvine Scientific, Tokyo, Japan). The medium was changed every 3 days. Human dermal fibroblasts (HDFs) were obtained from Cell Applications (San Diego, CA, USA) and cultured in Dulbecco’s modified Eagle’s medium (DMEM) (Thermo Fisher Scientific, Waltham, MA, USA) supplemented with 10% FBS (Thermo Fisher Scientific). The medium was changed every 3 days.

### Induction of neural crest cells (NCCs) from iPSCs

Human iPSCs were seeded onto iMatrix-511-coated plates or dishes at a density of 3.6 × 10^3^ cells/cm^2^ in StemFit AK03N medium and maintained in culture for 4 days. For NCC induction, the cells were cultured in StemFit Basic03 (equivalent to AK03N without bFGF, Ajinomoto, Tokyo, Japan) with 10 μM SB431542 (FUJIFILM Wako) and 1 μM CHIR99021 (Axon Medchem, Reston, VA, USA) for 10 days. Cell numbers were counted using a Countess II FL (Thermo Fisher Scientific). The medium was changed every 2 days from day 0 to 6 and every day from day 7 to 10.

### Differentiation of NCCs

For peripheral neuron and glial cell differentiation, 1 × 10^5^ CD271^high^ sorted NCCs were seeded onto fibronectin-coated 12-well plates and cultured in Neurobasal (Thermo Fisher Scientific) medium supplemented with B27 supplement (Thermo Fisher Scientific), N-2 supplement (Thermo Fisher Scientific), 2 mM L-glutamine (FUJIFILM Wako, Tokyo, Japan), 10 ng/mL BDNF (FUJIFILM Wako), GDNF (FUJIFILM Wako), NT-3 (FUJIFILM Wako), and NGF (FUJIFILM Wako) for 3 weeks. The medium was changed every 3 days. Differentiation was confirmed by immunostaining with peripherin, TUBB3, and GFAP.

For melanocyte differentiation, 2.5 × 10^5^ CD271^high^ sorted NCCs were seeded onto fibronectin-coated 12-well plates and cultured in Basic03 supplemented with 1 μM CHIR99021, 25 ng/mL BMP4 (R&D Systems, Minneapolis, MN, USA), and 100 nM Endothelin-3 (TOCRIS, Bristol, UK) for 10 days. The medium was changed every 2 days. Differentiation was confirmed by immunostaining with MITF.

### NCC expansion culture

CD271^high^ sorted NCCs were seeded onto fibronectin-coated plates at a density of 1 × 10^4^ cells/cm^2^ in Basic03 supplemented with 10 μM SB431542, 20 ng/mL EGF (FUJIFILM Wako), and FGF2 (FUJIFILM Wako). The medium was changed every 3 days. For passaging, cells were dissociated with Accutase (Innovative Cell Technologies, San Diego, CA, USA) and re-plated onto fibronectin-coated plates at a density of 1 × 10^4^ cells/cm^2^. For preparing the frozen stock of NCCs, 5 × 10^5^ NCCs were suspended in 500 µl STEM-CELL BANKER GMP grade (Takara, Kusatsu, Japan) and frozen using CoolCell Cell Freezing Containers (Biocision, Kyoto, Japan).

### Induction of mesenchymal stromal cells (XF-iMSCs) from NCCs

Expanded NCCs (passage number 4) were seeded onto fibronectin-coated plates at a density of 1 × 10^4^ cells/cm^2^ in Basic03 supplemented with 10 µM SB431542, 20 ng/mL EGF, and FGF2. The medium was replaced the next day with PRIME-XV MSC Expansion XSFM medium. The morphology of cells started to change approximately 4 days after induction. Passages were performed every 4 days using Accutase at a density of 1 × 10^4^ cells/cm^2^. Human MSC markers (CD44, CD73, CD90, and CD105) were analyzed by FACS 14 days after the MSC induction.

### Differentiation of XF-iMSCs

For chondrogenic differentiation, 1.5 × 10^5^ XF-iMSCs were suspended in 5 µl of chondrogenic medium (DMEM/F12, Thermo Fisher Scientific), 1% (v/v) ITS + premix (Corning, Corning, NY, USA), 0.17 mM AA2P (Sigma, St. Louis, MO, USA), 0.35 mM Proline (Sigma), 0.1 mM dexamethasone (Sigma), 0.15% (v/v) glucose (Sigma), 1 mM sodium-pyruvate (Thermo Fisher Scientific), 2 mM GlutaMAX (Thermo Fisher Scientific), and 0.05 mM MTG (Sigma) supplemented with 40 ng/mL PDGF-BB (PeproTech, Rocky Hill, NJ, USA), 100 ng/mL TGF-β3 (R&D), 10 ng/mL BMP4, and 1% (v/v) FBS (Thermo Fisher Scientific) and were subsequently transferred to fibronectin-coated 24-well plates. A total of 1 mL of chondrogenic medium was added after 1 h. The cells were cultured for 14 days. The differentiation properties of the cells were confirmed by Alcian blue staining. Briefly, induced cells were fixed for 30 min with 4% paraformaldehyde (PFA) (FUJIFILM Wako) and rinsed with phosphate buffered saline (PBS). These cells were then stained with Alcian Blue solution (1% Alcian Blue (MUTO PURE CHEMICAL CO., LTD, Tokyo, Japan) for 1 h at 25 °C.

For osteogenic differentiation, 4 × 10^4^ XF-iMSCs were seeded onto 0.1% gelatin-coated 12-well plates and cultured in MSCgo Rapid Osteogenic Differentiation Medium (Biological Industries, Cromwell, CT, USA) for 30 days. The medium was changed every 3 days. Differentiation properties were confirmed by the formation of calcified nodules, as detected with Alizarin Red (Merck, Darmstadt, Germany) staining. Briefly, culture wells were washed twice in PBS and fixed for 10 min at room temperature in 100% ethyl alcohol. The Alizarin Red solution (40 mM, pH 4.2) was applied to the wells for 10 min at room temperature. Nonspecific staining was removed by several washes with water.

For adipogenic differentiation, 4 × 10^4^ XF-iMSCs were seeded onto a 0.1% gelatin-coated 12-well plate and cultured in hMSC Adipogenic Differentiation Medium (Lonza, Basel, Switzerland) for 32 days. The medium was changed every 3 days. Differentiation properties were confirmed by Oil Red O staining. The cells were fixed in 10% formalin for 1 h at room temperature, followed by incubation for 20 min in 0.3% Oil Red O staining solution (Sigma). Nonspecific staining was removed by performing several washes with water.

### IFN-γ primed MSCs

XF-iMSCs and human adult-derived MSCs were treated with 10 ng/mL IFN-γ (Peprotech) for 24 h. After 24 h, the cells were collected, and RT-qPCR was performed. PGE2 measurement of MSC’s conditioned medium was measured using the PGE2 Express ELISA Kit (Cayman chemical, MI, USA) according to the manufacturer’s protocol. Briefly, standards and samples were incubated with PGE2 antibody in 96-well plate for 60 min at 25 °C. The plate was washed by 0.05% Tween 20 contained wash buffer. After wash, Ellman’s Reagent which contains the substrate to AChE is added to the well and incubate 60 min at 25 °C (Protect from light). Read the plate at wavelength 420 nm by microplate reader Spark (Tecan, Zurich, Switzerland).

### Immunocytochemistry

Prior to performing immunostaining with antibodies, the cells on plates were fixed with 4% PFA/PBS (FUJIFILM Wako) at 4 °C for 15 min, washed twice with PBS, and incubated with 0.3% TritonX100 at 4 °C (as the surface-active agent for penetration processing) for 30 min, and any nonspecific binding was blocked with 3% BSA/PBS at 4 °C for 1 h. DAPI (1:1000; Thermo Fisher Scientific) was used to counterstain the nuclei. The primary antibodies used in this study are summarized in Supplementary Table 1. Observations and assessments of samples were performed with BZ-X700 (Keyence, Osaka, Japan).

### FACS sorting

FACS was performed using an AriaII instrument (BD Biosciences, Franklin Lakes, NJ, USA) according to the manufacturer’s protocol. Briefly, cells were dissociated by Accutase and incubated with any antibodies in FACS buffer (2% human serum albumin containing HBSS) and incubated for 60 min at 4 °C. The antibodies used are listed in Supplementary Table 1. In all experiments, an isotype control was used to remove the nonspecific background signal. After the antibody reaction, cells were washed by FACS buffer and re-suspend to 0.1% Propidium iodide (PI) containing FACS buffer. Finally, cells were collected by passing through a 35 μm filter (Falcon) and analyzed by FACS AriaII. Gating strategies indicated in Supplementary Fig. 14. FCM data were analyzed by FlowJo software (BD) according to the manufacturer’s protocol.

### Quantitative RT-PCR

Total RNA was purified using the RNeasy Mini Kit (Qiagen, Valencia, CA, USA) and treated with the DNase-one Kit (Qiagen) to remove genomic DNA. We reverse transcribed 500 ng of total RNA to obtain single-stranded cDNA using PrimeScript RT Master Mix (Takara) according to the manufacturer’s instructions. Quantitative PCR with the Thunderbird SYBR qPCR Mix (TOYOBO, Osaka, Japan) was performed using the QuantStudio 7 Flex Real-Time PCR System (Applied Biosystems, Forester City, CA, USA) in triplicate. Primer sequences are listed in Supplementary Table 2. *IDO1* (Hs00984148_m1) and *PD-L1* (Hs00204257_m1) expressions were measured by TaqMan Gene Expression Assays (Thermo Fisher Scientific).

### Transcriptome analysis

Total RNA was purified using the RNeasy Micro Kit (Qiagen) and treated using the DNase-one kit (Qiagen) to remove genomic DNA. We reverse transcribed 10 ng of total RNA to obtain single-stranded cDNA using the SuperScript VILO cDNA Synthesis Kit (Thermo Fisher Scientific). We performed cDNA library synthesis for the Ion Ampliseq transcriptome using the Ion AmpliSeq Transcriptome Human Gene Expression Core Panel (Thermo Fisher Scientific) and the Ion Ampliseq Library Kit Plus (Thermo Fisher Scientific) according to the manufacturer’s protocol. Briefly, cDNA was amplified 12 cycles with Ion AmpliSeq^TM^ Transcriptome Human Gene Expression Core Panel by thermal cycler. Primer sequences were partially digested with FuPa reagent by sequentially performing 10 min at 50 °C, 10 min at 55 °C, 20 min at 60 °C. Barcode ligation was performed with Ion Xpress Barcode for 30 min at 22 °C. Barcode-labeled cDNA libraries were purified by DNA Clean & Concentrator™-5 (Zymo research, CA, USA) and analyzed using the Ion S5 XL System (Thermo Fisher Scientific) and the Ion 540 Chip Kit (Thermo Fisher Scientific).

### Preparation of XF-C-iMSCs

XF-C-iMSCs were prepared as reported previously, with minor modifications^45^. Briefly, XF-iMSCs were seeded at a density of 1.0 × 10^5^ cells/well in 48-well plates (Corning, Corning, NY, USA) coated with fibronectin and cultured with Prime-XV MSC expansion XSFM for 4 days. To obtain XF-C-iMSCs, confluent cells that had formed on the cellular sheet consisting of the ECM produced by MSCs themselves were scratched using a micropipette tip and then torn off. The iMSC/ECM complexes detached from the bottom of the plate in a sheet shape were transferred to a 24-well ultra-low binding plate (Corning) and rolled up to form a round clump of cells. The cell clumps were maintained in Prime-XV MSC expansion XSFM or MSCgo Osteogenic differentiation medium (Biological Industries) for 2, 5, or 10 days.

### Staining of XF-C-iMSCs

XF-C-iMSCs were fixed with 4% PFA in PBS. The samples were embedded in paraffin, and 5 μm thick serial sections were prepared. The specimens were then stained with hematoxylin and eosin (H&E) or Alizarin Red S and observed using a Nikon Eclipse E600 microscope (Nikon, Kawasaki, Japan).

### Surgical procedures

Male NOD/SCID mice (7 to 8-week-old) (Charles River Laboratories Japan) were employed as a calvarial defect model after ethical approval was obtained from the Animal Care Committee of Hiroshima University (A18-72). Surgery was performed under general anesthesia, with an intraperitoneal injection of 20% ethyl carbamate (30 mg/kg body weight). The skin at the surgical site was shaved and disinfected, and a sagittal skin incision was made from the occipital to the frontal bone. The skin flap, including the periosteum, was then dissected and elevated. Avoiding the sagittal and coronal suture, a 1.6-mm diameter defects was created in the lateral parietal bone 4 mm lateral and 3 mm posterior to the bregma. XF-C-iMSCs cultured with MSCgo osteogenic differentiation medium for 2 days were transplanted into the defect with no artificial scaffold. In addition, the implantation of XF-C-BMMSCs maintained in the same medium was used as a control. The skin incision was then closed using 4-0 silk sutures.

### Micro-CT analysis

Mice were sacrificed 28 days after surgery, and the cranial region was imaged using a SkyScan1176 in vivo micro-CT (Bruker, Billerica, MA, USA). Three-dimensional reconstructions were generated using the CTVOL software (Bruker). The volume of newly formed bone inside the bone defect was determined using CT-An software (Bruker).

### Tissue preparation and histological analysis of the skull

The mice were sacrificed 28 days after surgery. Calvarial bones were collected, fixed with 4% PFA overnight, and decalcified with 10% (v/v) ethylenediaminetetraacetic acid (pH 7.4) for 10 days. After decalcification, the specimens were dehydrated through graded ethanol, cleared with xylene, and embedded in paraffin. Serial sections (5 μm) were cut in the frontal plane. These sections representing the central portion of the bone defect were stained with H&E and observed using a Nikon Eclipse E600 microscope. For Azan staining, the slides were incubated in mordant solution (a mixture of equal parts of 10% potassium dichromate and 10% trichloroacetic acid) for 10 min and washed with H_2_O.

The slides were then incubated in azocarmine G solution (0.1% azocarmine G, 1% acetic acid) for 30 min. The specimens were briefly washed with H_2_O, differentiated with anilin alcohol (0.1 mL anilin dissolved in 100 mL 95% ethanol), washed with acetic alcohol and H_2_O, incubated in phosphotungstic acid (5%) for 1 h, briefly washed with H_2_O, incubated in aniline blue/orange G solution (0.5% anilin blue, 2% orange G, 8% acetic acid) for 30 min, and then briefly washed with H_2_O. Subsequently, the slides were incubated in 100% alcohol and xylene before they were embedded using mounting medium. To detect human vimentin expression in the tissue, immunofluorescence analysis was performed. Briefly, serial sections (20 μm) were blocked with 1% BSA/0.1% Triton-X/PBS blocking solution at room temperature for 30 min. These sections were then incubated with rabbit anti-human vimentin monoclonal IgG antibody (Abcam; #SP20) at 4 °C overnight. After washing with PBS three times for 5 min, the samples were incubated for 1 h with an Alexa Fluor 488^®^ goat anti-rabbit IgG antibody (Thermo Fisher Scientific) at room temperature. Nuclei were counterstained with DAPI. Fluorescence signals were detected using a Zeiss LSM 510 laser scanning confocal microscope (Zeiss, Oberkochen, Germany).

### Skeletal muscle injury and transplantation

Ethical approval for these experiments was obtained from the Animal Care Committee of Kyoto University (14-59-8). For the transplantation of XF-iMSCs, 8 - to 16-week-old NSG mice were purchased from Charles River Japan (Yokohama, Japan). Mice were anesthetized with 3% Forane inhalant liquid (AbbVie, North Chicago, IL, USA). The midportion of the TA muscle was then continuously crushed by direct clamping with forceps for 1 min under constant pressure determined using the same pressure gauge^46^. XF-iMSCs or HDFs were suspended in αMEM (2 × 10^5^ cells/50 µL) and injected using a 27 G micro-syringe at the center of the injured sites of the TA muscles 24 h post injury.

### Tissue preparation and histological analysis of muscle

Mice were sacrificed 3 days, 2 weeks, and 5 weeks post injury. TA muscles were mounted in Tragacanth Gum (FUJIFILM Wako) and frozen with liquid nitrogen^47^. Serial sections (10 µm) were cut using a cryostat. Sections were stained with H&E and observed using an Olympus BX51 microscope (Olympus, Tokyo, Japan). Four sections prepared from the middle of each TA muscle samples were stained, and the entire transverse section of all sections was photographed and analyzed. Of the values obtained from the four thin sections taken, the one with the highest value was adopted as the data for each sample. Immunofluorescence images were acquired using the Zeiss LSM 710 laser scanning confocal microscope (Zeiss). Area measurement and cell counting were performed using Hybrid cell count software BZ-H3C (Keyence). In Fig. 6C, images stained with Laminin antibody were analyzed with KEYENCE image analysis software to calculate the average area of myofibers. In Fig. 6E, images stained with MYH4 antibody were analyzed with KEYENCE image analysis software to calculate the area of the positive area. In Fig. 6G, images stained with MYH3 antibody were counted for the number of positive fibers.

### Myotube differentiation from mouse newborn myoblasts

Ethical approval for these experiments was obtained from the Animal Care Committee of Shonan Health Innovation Park (AU-00020995). Myoblasts were isolated from newborn C57BL/6 mice (CLEA Japan, Tokyo), as described previously^48^. Myoblasts were cultured on 10% Matrigel (Corning)-coated dishes in high glucose DMEM with 20% FCS, 10% horse serum, 0.5% chicken embryo extract, 2.5 ng/mL FGF2, 10 μg/mL gentamycin, 1% antibiotic-antimitotic, and 2.5 μg/mL plasmocin prophylaxis (Proliferation Medium: PM). For myotube differentiation, 1 × 10^5^ myoblasts were seeded onto 10% Matrigel-coated 24-well plates and cultured in high glucose DMEM with 5% horse serum and 1% antibiotic-antimitotic (Differentiation Medium: DM) for 3 days. XF-iMSC-cultured medium was obtained by incubating XF-iMSCs in DM medium for 48 h. Human PXDN recombinant protein (Abnova, Taipei, Taiwan) was used at 0.5 μM according to a previous report^49^. Human IGF2 recombinant protein (R&D) was used at 300 ng/mL. Myotube movement was analyzed using the ImageJ software with the TPIV plugin (https://signaling.riken.jp/tools/imagej-plugins/490/).

### Quantification of secreted proteins in cultured media

Cultured media were obtained by incubating XF-iMSCs, HDFs, hAC-MSCs, hBM-MSCs, and hUC-MSCs in PRIME-XV MSC Expansion XSFM medium for 48 h. Albumin and immunoglobulin were removed from the cultured media using High select top14 abundant protein depletion mini spin columns (Thermo Fisher Scientific). The depleted medium was precipitated by acetone and subjected to trypsin digestion followed by desalting. These samples were labeled with mTRAQ reagent (AB Sciex Co. Ltd., Toronto, Canada) and analyzed on an Orbitrap fusion tribrid mass spectrometer coupled with an Easy n-LC 1000 HPLC system (Thermo Fisher Scientific). The expressed proteins were identified by matching the peptide data obtained by LC-MS with data obtained through Mascot search (Proteome discoverer 1.4). (Thermo Fisher Scientific). The search conditions in the Mascot search were follows. Database: Swiss prot_Human, Enzyme: Trypsin, Miss cleave: <3, MS tolerance: MS < 10 ppm, MS/MS < 0.35 Da, Modification: Daynami, Oxidation(M), mTRAQ(N-Term/K); Static, Carbamidomethyl(C).

### Reporting summary

Further information on research design is available in the Nature Research Reporting Summary linked to this article.

## Supplementary information

supplementary_movie_1

supplementary_movie_2

supplementary_movie_3

supplementary_movie_4

supplementary_information

REPORTING SUMMARY

## Data Availability

RNA-seq data that support the findings of this study have been deposited in the Gene Expression Omnibus (GEO) database with the following accession code GSE206048, GSE206128, GSE206172. Proteome data that support the findings of this study have been deposited in Japan Proteome Standard Repository (jPOSTrepo) database with the following accession code JPST001693.

## Code availability

TPIV plugin for Image J software is available from Riken research resource page (https://signaling.riken.jp/tools/imagej-plugins/490/).
