# Supplementary material for: Induction of functional xeno-free MSCs from human iPSCs via a neural crest cell lineage
Source: NPJ Regen Med. 2022 Sep 15;7:47. doi: 10.1038/s41536-022-00241-8 (PMC9477888; doi:10.1038/s41536-022-00241-8)
Supplement: Supplementary file 5 — supplementary_information [file 41536_2022_241_MOESM5_ESM.pdf]

Supplementary Figure 1

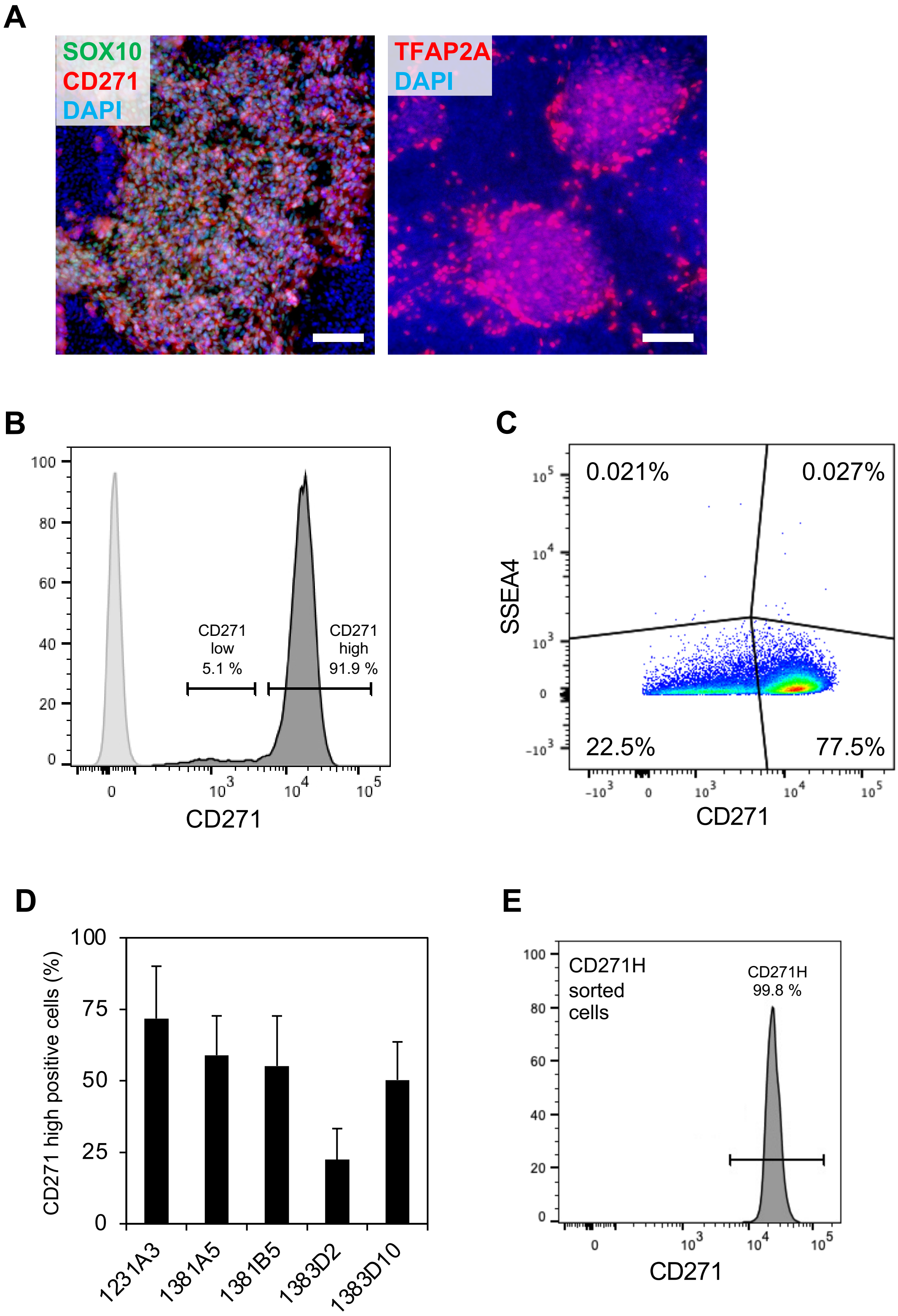

Supplementary Figure 1. Successful induction of iNCCs from iPSCs

(A) Immunofluorescence images of the NCC induction at day 10. Cells were stained with anti-SOX10 antibody (left panel, green), anti-CD271 antibody (left panel, red), and anti-TFAP2A antibody (right panel, red). Nuclei were stained with DAPI (blue). Scale bar, 50 μm. (B) The fraction of cells with high CD271 expression (CD271H) on day 10 of NCC induction (dark gray) of 1231A3 iPSCs. The isotype control is gray. (C) A dot plot analysis of the NCC induction at day 10. The x-axis indicates CD271 expression, and the y-axis indicates SSEA4 expression. (D) Fraction of the CD271H population on day 10 of the NCC induction of various iPSC lines (1231A3, 1381A5, 1381B5, 1382D2, 1383D10). Data are the mean ± SD, n = 3. (E) The fraction of CD271H cells after sorting.

Supplementary Figure 2

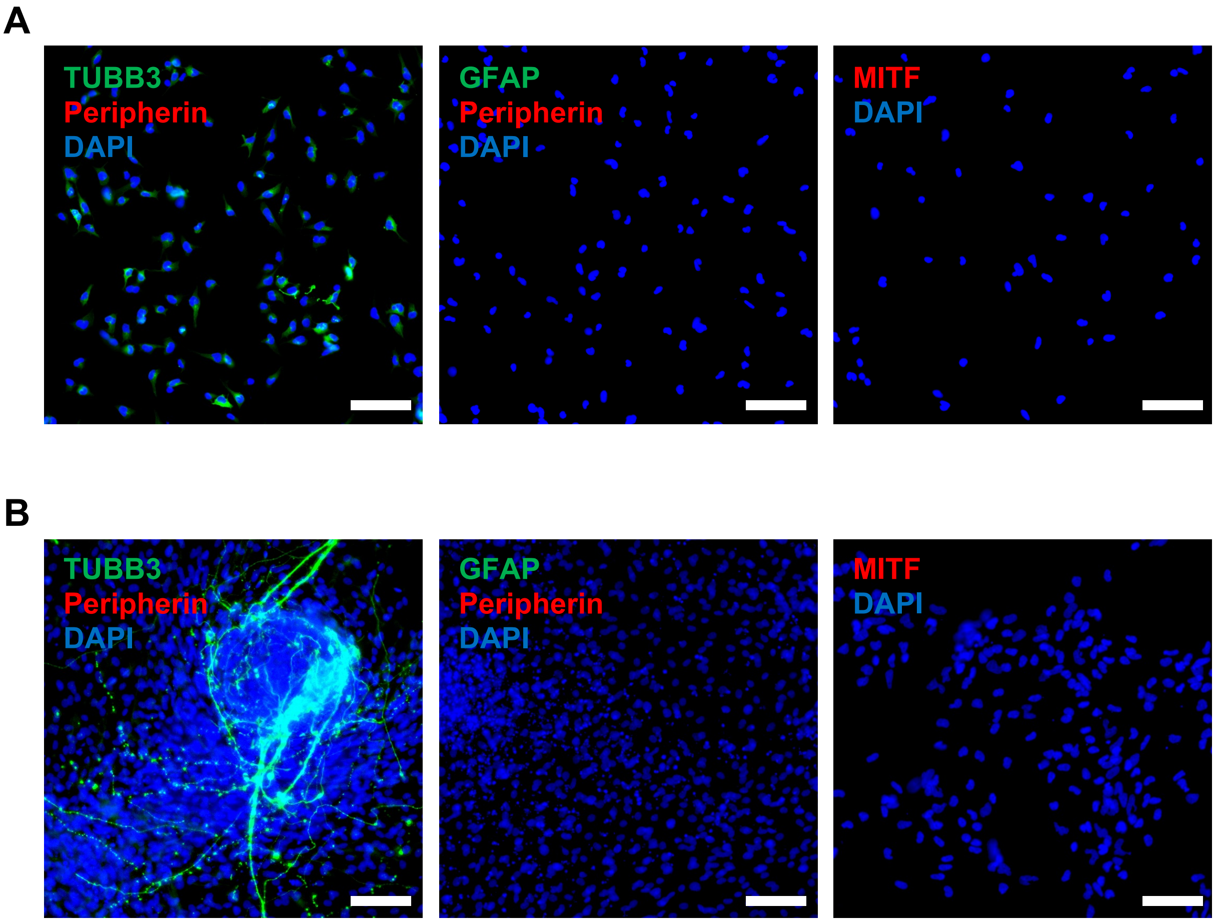

Supplementary Figure 2. Characteristics of CD271H and CD271L sorted cells.

Immunofluorescence images of (A) CD271H sorted cells and (B) CD271L sorted cells at day 10 of the NCC induction. Left panel, cells were stained with an anti-TUBB3 antibody (green) and anti-peripherin antibody (red); middle panel, cells were stained with an anti-GFAP antibody (green) and anti-peripherin antibody (red); right panel, cells were stained with an anti-MITF antibody (red). Nuclei were stained with DAPI (blue). Scale bars, 50 µm.

Supplementary Figure 3

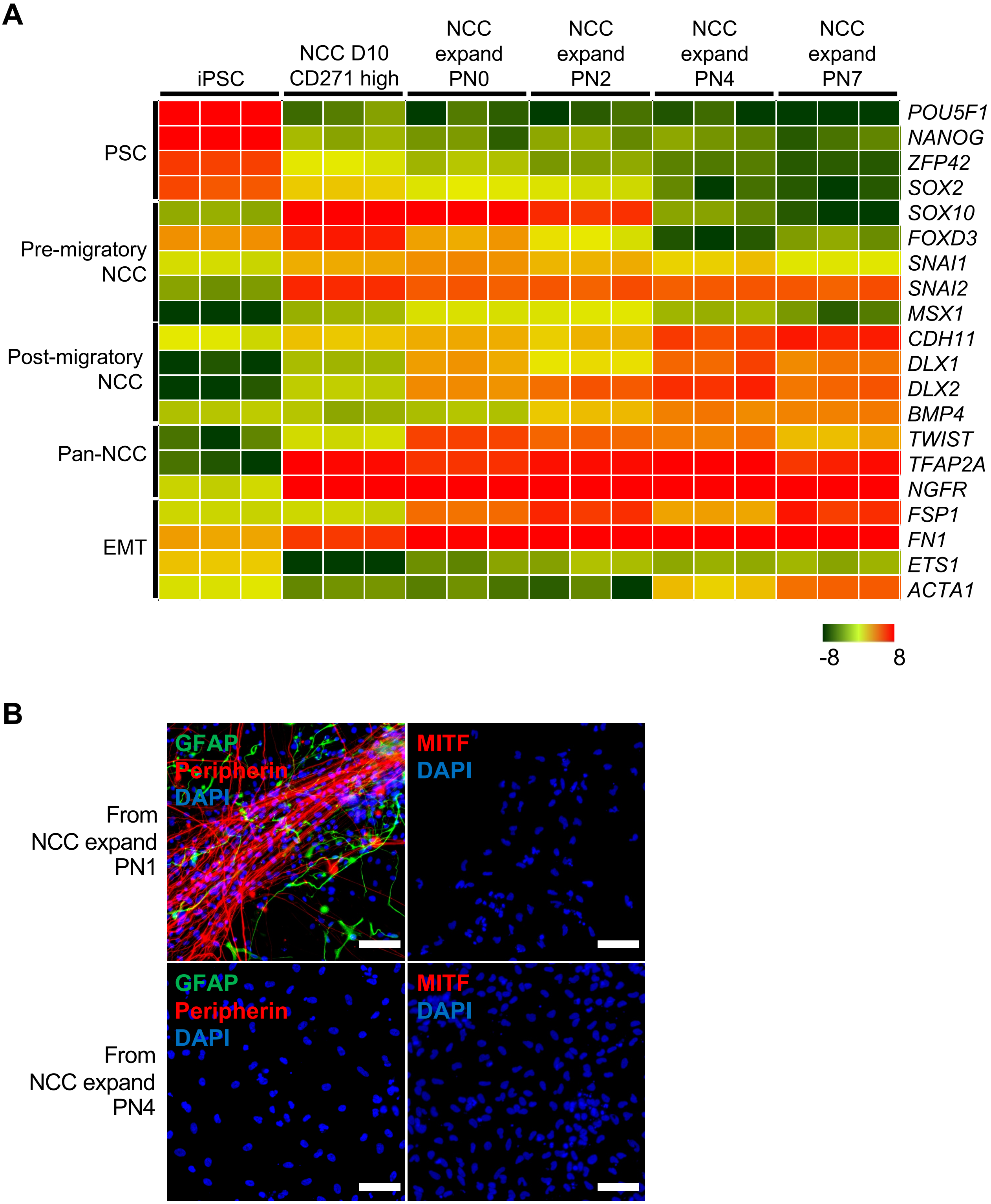

Supplementary Figure 3. Gene expression pattern and differentiation ability in NCC expansion culture

(A) A heatmap illustrating the expression of pluripotent stem cell (PSC), pre-migratory NCC, post-migratory NCC, Pan-NCC and epithelial mesenchymal transition (EMT) marker genes for 1231A3 iPSCs, CD271H population on NCC induction at day10, and NCC expansion culture passage number (PN) 0, 2, 4, and 7. All samples were n = 3. (B) Immunofluorescence images of neural (left) and melanocyte (right)-inducing cells from NCC expansion culture PN1 (upper panel) and PN4 (lower panel). Left panel, cells were stained with an anti-GFAP antibody (green) and anti-peripherin antibody (red); right panel, cells were stained with an anti-MITF antibody (red). Nuclei were stained with DAPI (blue). Scale bars, 50 μm. All sequence data were available in GEO (GSE206128)

Supplementary Figure 4

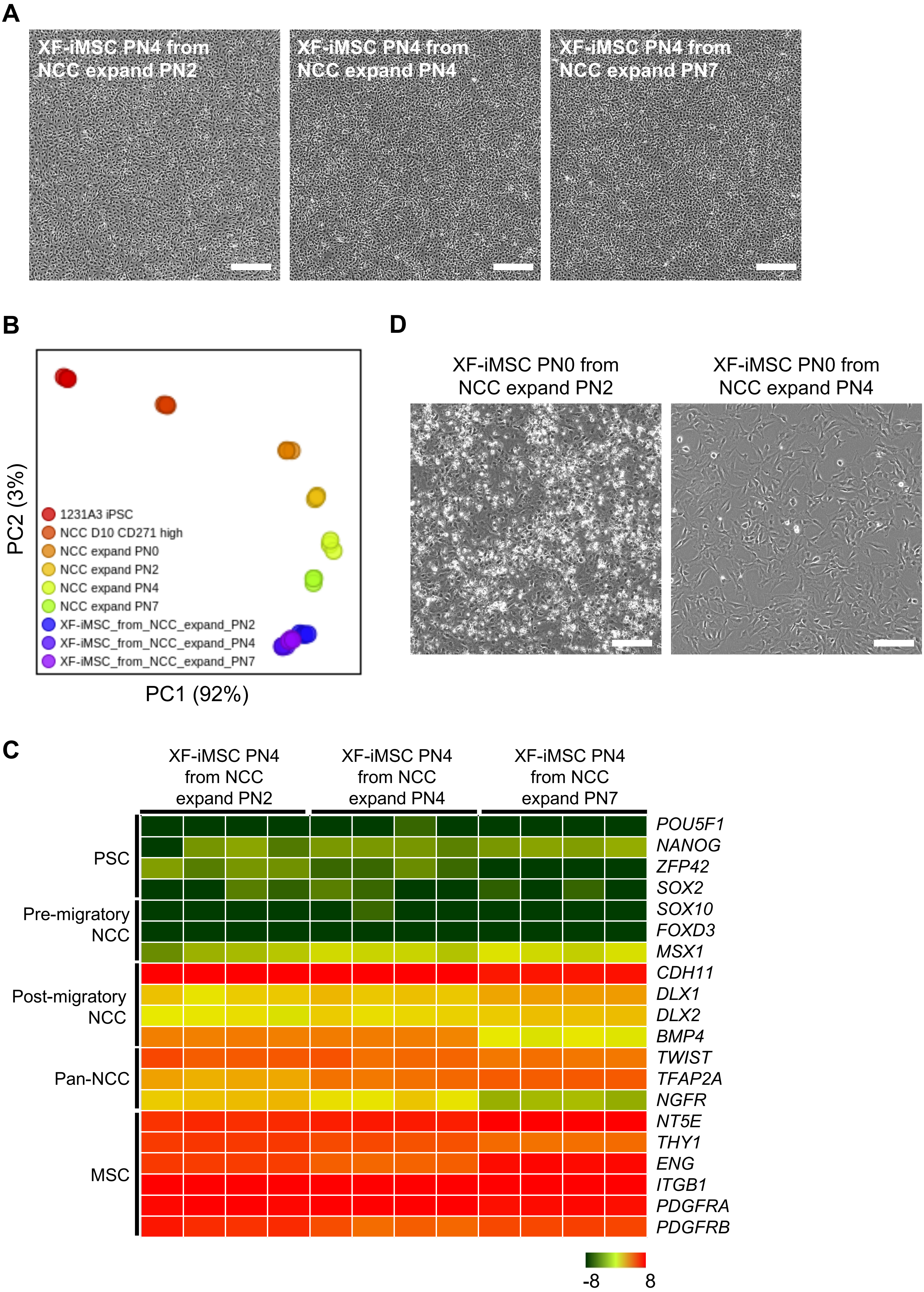

Supplementary Figure 4. MSC induction and characterization of different NCC expansion culture passage numbers

(A) Phase contrast images of XF-iMSC passage number (PN) 4 from NCC expansion culture PN2 (left), PN4 (middle) and PN7 (right). Scale bars, 200 µm. (B) PCA analysis of the MSC induction from different NCC expansion culture. PC1: principal component 1, PC2: principal component 2. (C) Heatmap illustrating the expression of pluripotent stem cell (PSC), pre-migratory NCC, post-migratory NCC, Pan-NCC and MSC marker genes for MSCs from NCC expansion culture PN2, 4, 7. All samples were n = 4. (D) Phase contrast images of XF-iMSC PN0 from NCC expansion culture PN2 (left) or PN4 (right). Scale bars, 100 µm. All sequence data were available in GEO (GSE206128)

Supplementary Figure 5

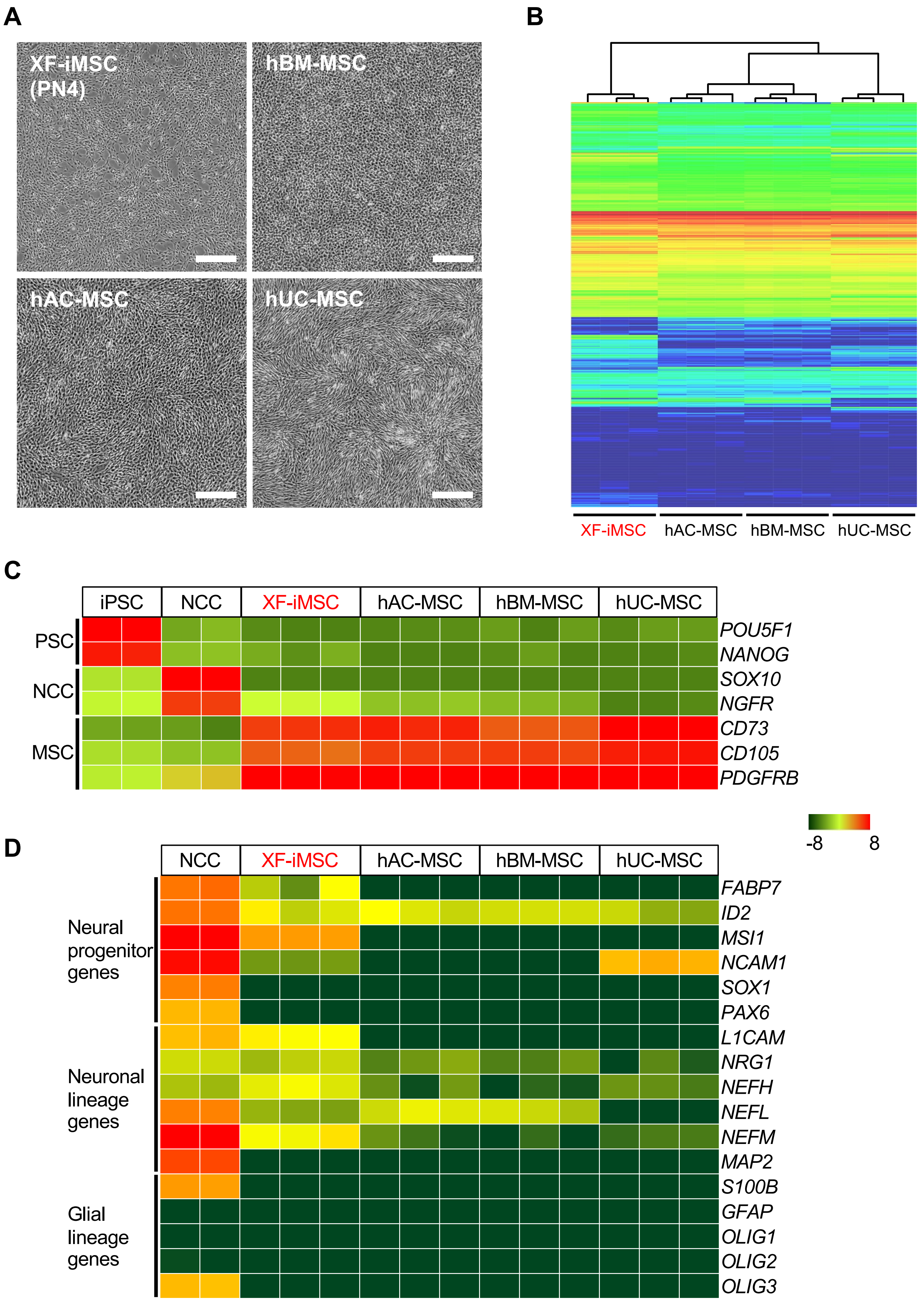

Supplementary Figure 5. Xf-iMSCs have similar characteristics to adult-derived MSCs.

(A) Phase contrast images of XF-iMSCs passage number (PN) 4, human adipocyte-derived mesenchymal stromal cells (hAC-MSCs), human bone marrow-derived mesenchymal stromal cells (hBM-MSCs), and human umbilical cord-derived MSCs (hUC-MSCs). Scale bar, 200 μm. (B) Hierarchical clustering analysis of XF-iMSCs, hAC-MSCs, hBM-MSCs, and hUC-MSCs. n = 3. (C) Heatmap illustrating the expression of PSC, NCC, MSC genes for 1231A3 iPSCs, induced NCCs on day 10 (NCC), XF-iMSCs, hAC-MSCs, hBM-MSCs, and hUC-MSCs. n = 2 (iPSCs, NCCs), n = 3 (XF-iMSCs, hAC-MSCs, hBM-MSCs, hUC-MSCs). (D) Heatmap illustrating the expression of neural progenitor, neuronal lineage, and glial lineage genes for NCC induction on day 10 (NCCs), XF-iMSCs, hAC-MSCs, hBM-MSCs, and hUC-MSCs. N = 2 (NCC), N = 3 (XF-iMSCs, hAC-MSCs, hBM-MSCs, hUC-MSCs). All sequence data were available in GEO (GSE206172).

Supplementary Figure 6

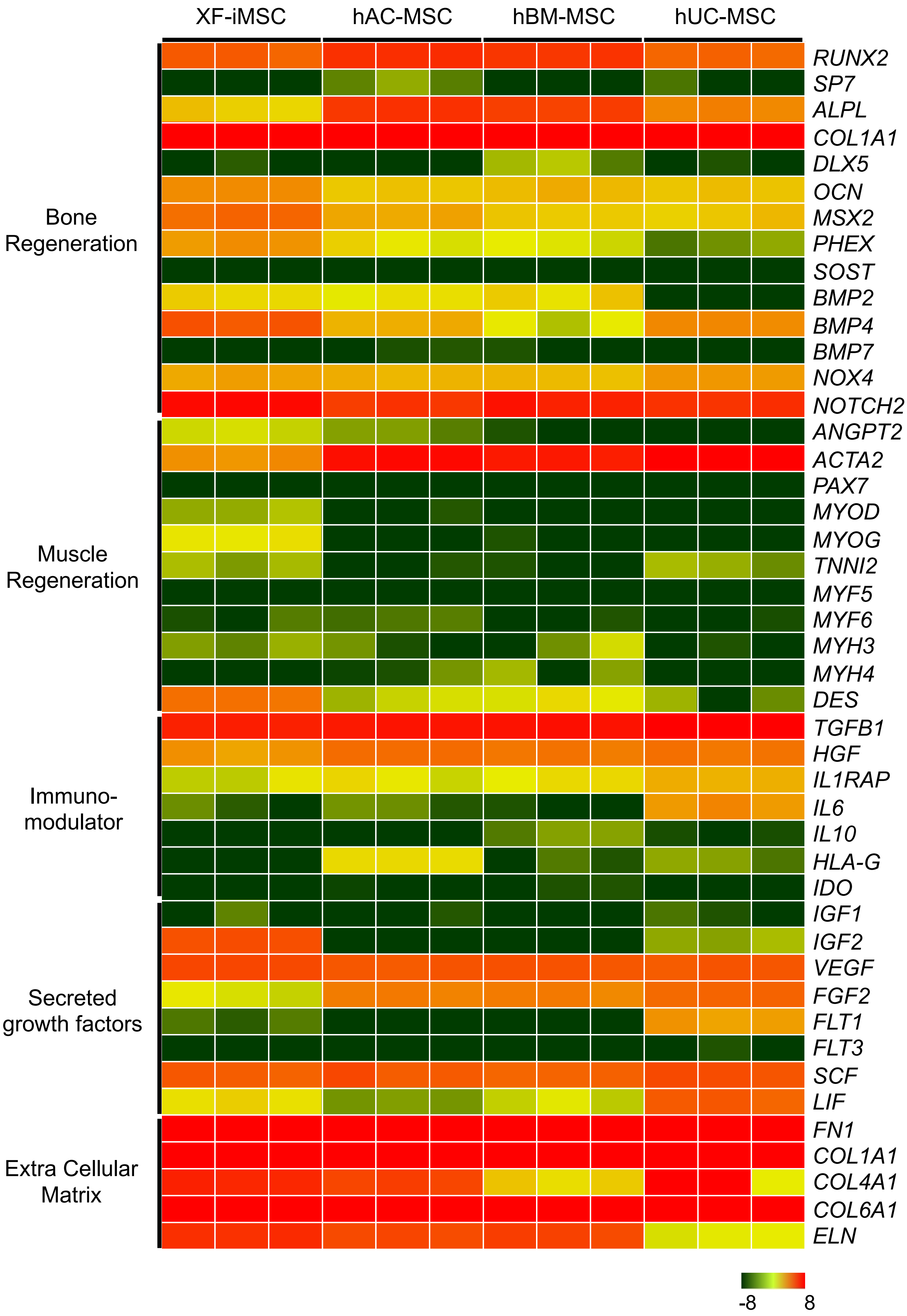

Supplementary Figure 6. Gene expression patterns in XF-iMSCs and human adult derived MSCs

A heatmap illustrating the expression of bone regeneration, muscle regeneration, immunomodulator, secreted growth factors and extra cellular matrix genes for XF-iMSCs, hAC-MSCs, hBM-MSCs and hUC-MSCs. All samples were n = 3. All sequence data were available in GEO (GSE206172).

Supplementary Figure 7

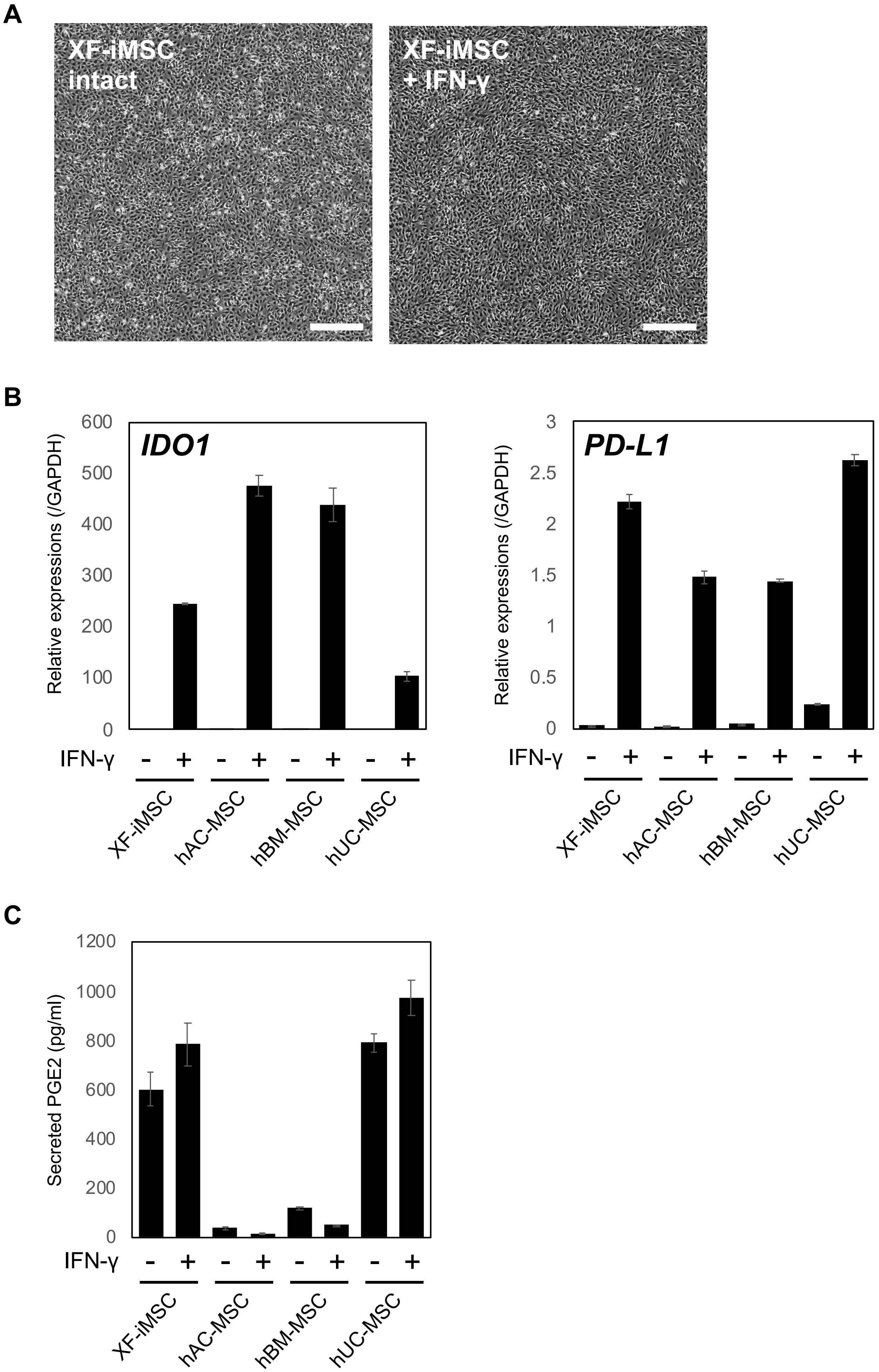

Supplementary Figure 7. IFN-γ primed MSCs

(A) Phase contrast images of XF-iMSCs (left) treated with 10 ng/mL IFN-γ (right) for 24 hours. Scale bars, 200 μm. (B) The mRNA expression of IDO1 (left) and PD-L1 (right) in XF-iMSCs and human adult-derived MSCs with or without 10 ng/mL IFN-γ for 24 hours. The mRNA expression of each gene was analyzed using RT-qPCR. Data are the mean ± SD, n = 3. (C) PGE2 secretion from XF-iMSCs and human adult derived MSCs with or without 10 ng/m L IFN-γ for 24 hours. Data are the mean ± SD, n = 3.

Supplementary Figure 8

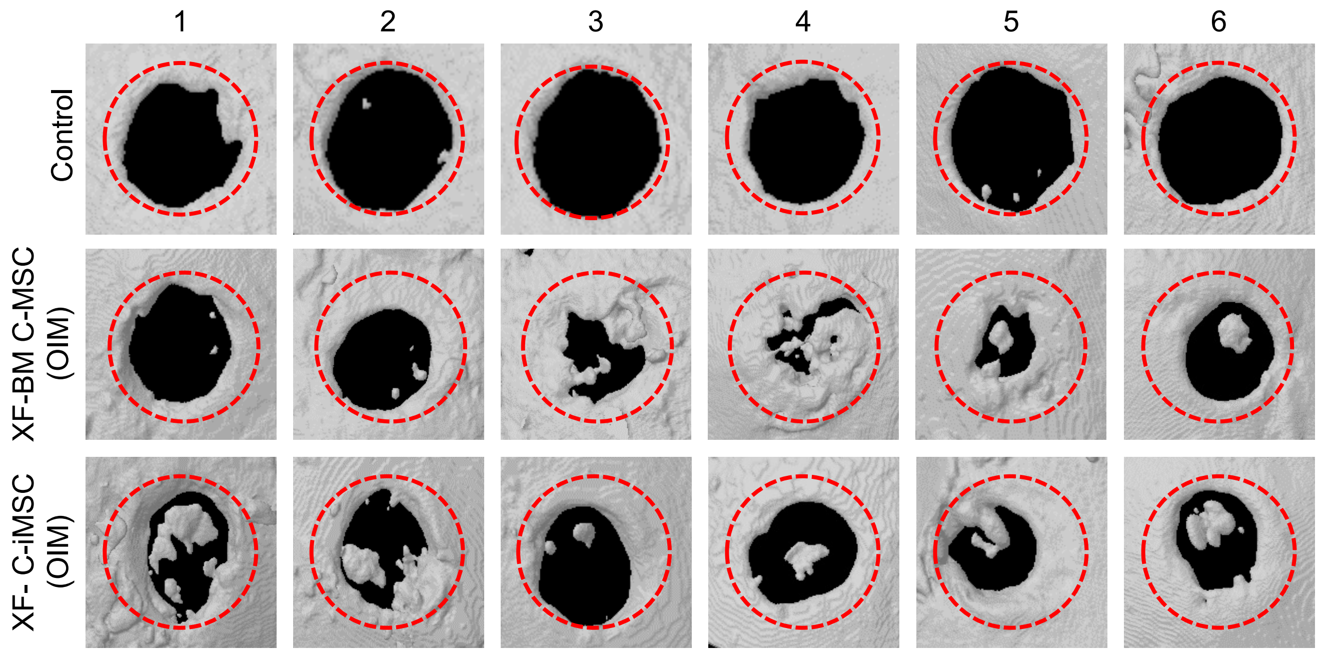

Supplementary Figure 8. Skull bone regeneration by transplanted clump-hBM-MSCs or clump-XF-iMSCs generated from osteogenic induction medium (OIM)

Individual CT-scanning images of the skulls of control (upper panel), hBM-C-MSCs (middle panel) and XF-C-MSCs-transplanted mice. All samples were n = 6.

Supplementary Figure 9

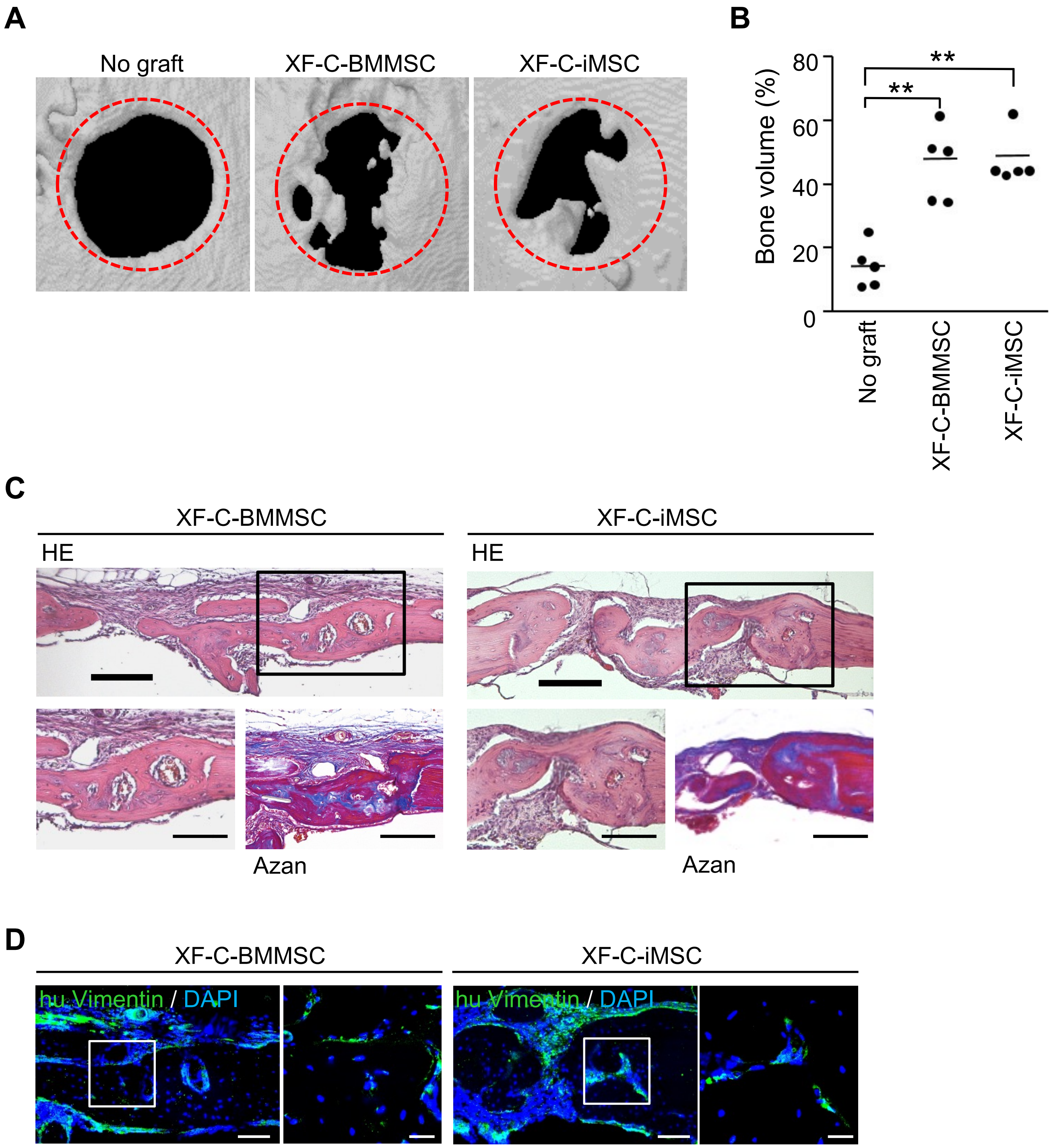

Supplementary Figure 9. Enhanced skull bone regeneration by transplanted clump-XF-iMSCs.

(A) CT-scanning images of the skulls of transplanted mice. Trepanned areas are indicated by the red circles. (B) Relative bone volumes of No graft, XF-C-BMMSCs, and XF-C-iMSCs. Data are mean ± SD, n = 5. $^{**}P < 0.01$. (C) Lateral section images of the transplanted skulls. Sections were stained by HE and Azan (right bottom). Black boxes in (C) have the same areas as those of the serial sections in (D). Scale bars, 500 μm. (D) Lateral section fluorescence images of transplanted skulls. Sections were stained by anti-human vimentin (green). Nuclei were stained with DAPI (blue). The right column shows high magnification images of the white box in the left column. Scale bar, 100 μm (left column) and 10 μm (right column).

Supplementary Figure 10

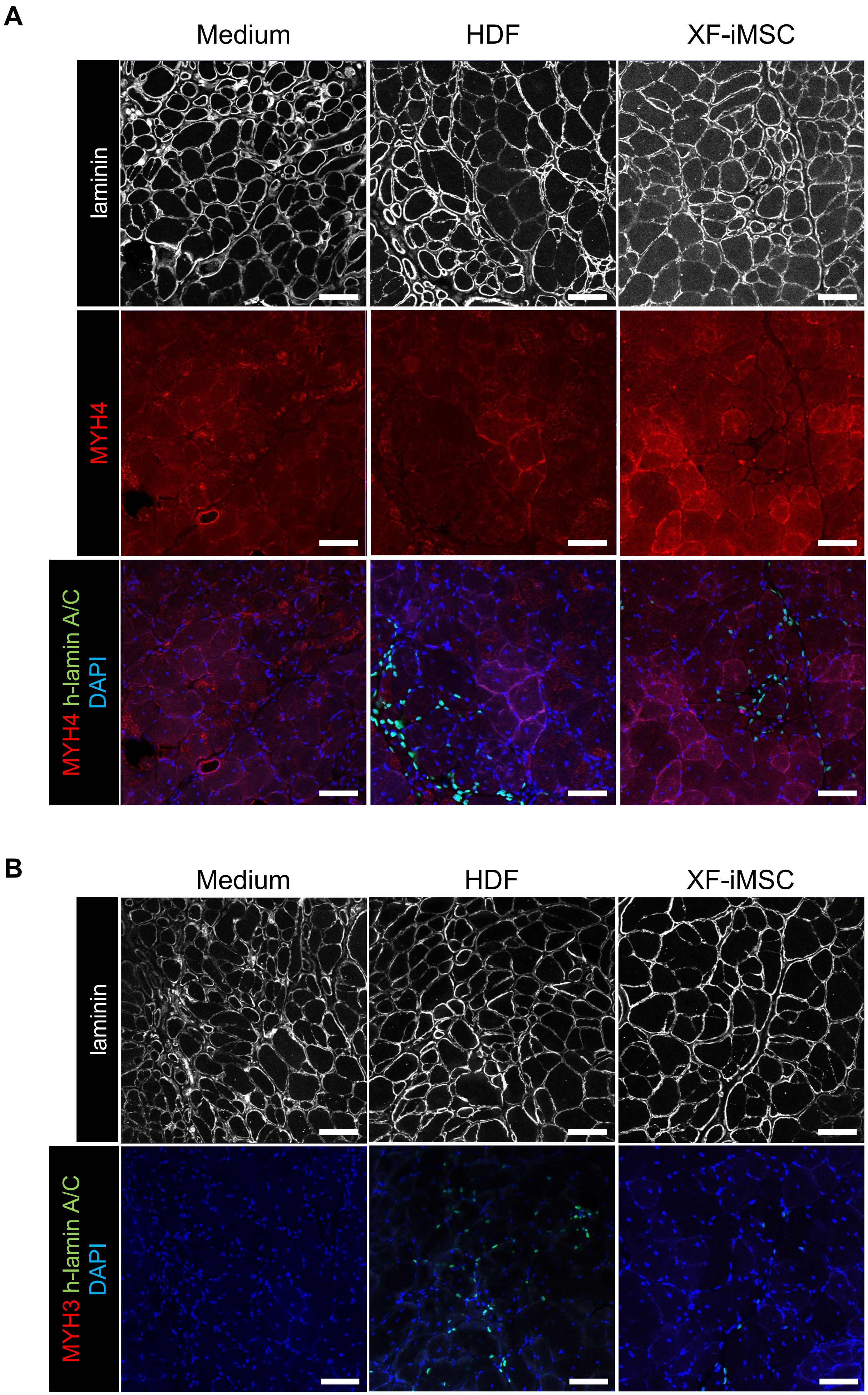

Supplementary Figure 10. Muscle regeneration by transplanted XF-iMSCs

(A) Sectional images of injured TA muscle 5 weeks after transplantation. Sections were stained with laminin (white), MYH4 (red) and MYH4 (red) / human lamin A/C (green) antibodies. Nuclei were stained with DAPI (blue). Scale bars, 100 μm. (B) Sectional images of injured TA muscle 5 weeks after transplantation. Sections were stained with laminin (white), MYH3 (red) / human lamin A/C (green) antibodies. Nuclei were stained with DAPI (blue). Scale bars, 100 μm.

Supplementary Figure 11

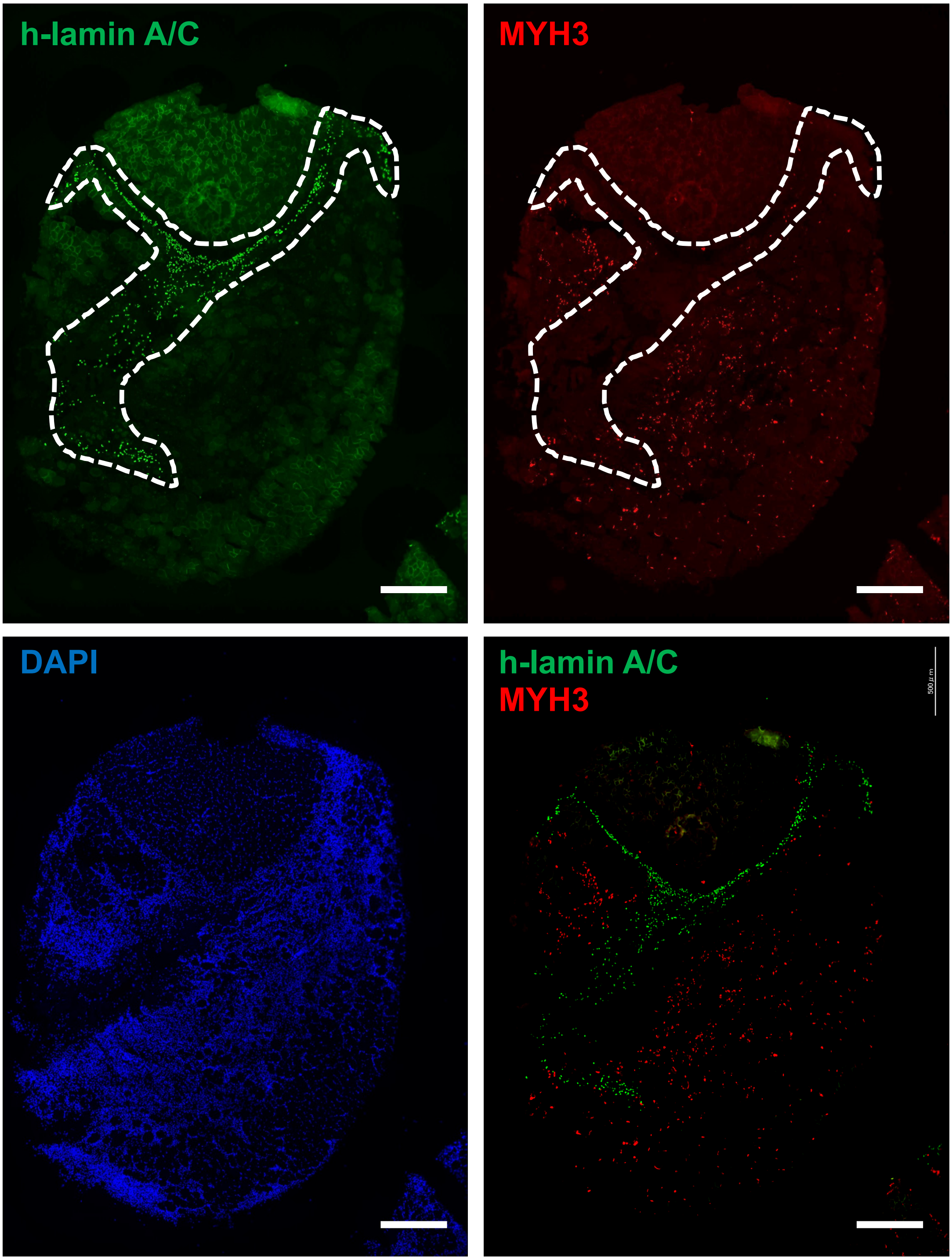

Supplementary Figure 11. Most MYH3-positive cells are found around transplanted XF-iMSCs

Sectional images of injured TA muscle 3 days after XF-iMSCs transplantation. Sections were stained with human lamin A/C (green), MYH3 (red) antibodies. Nuclei were stained with DAPI (blue). White dotted lines indicate the transplanted area. Scale bars, 500 μm.

Supplementary Figure 12

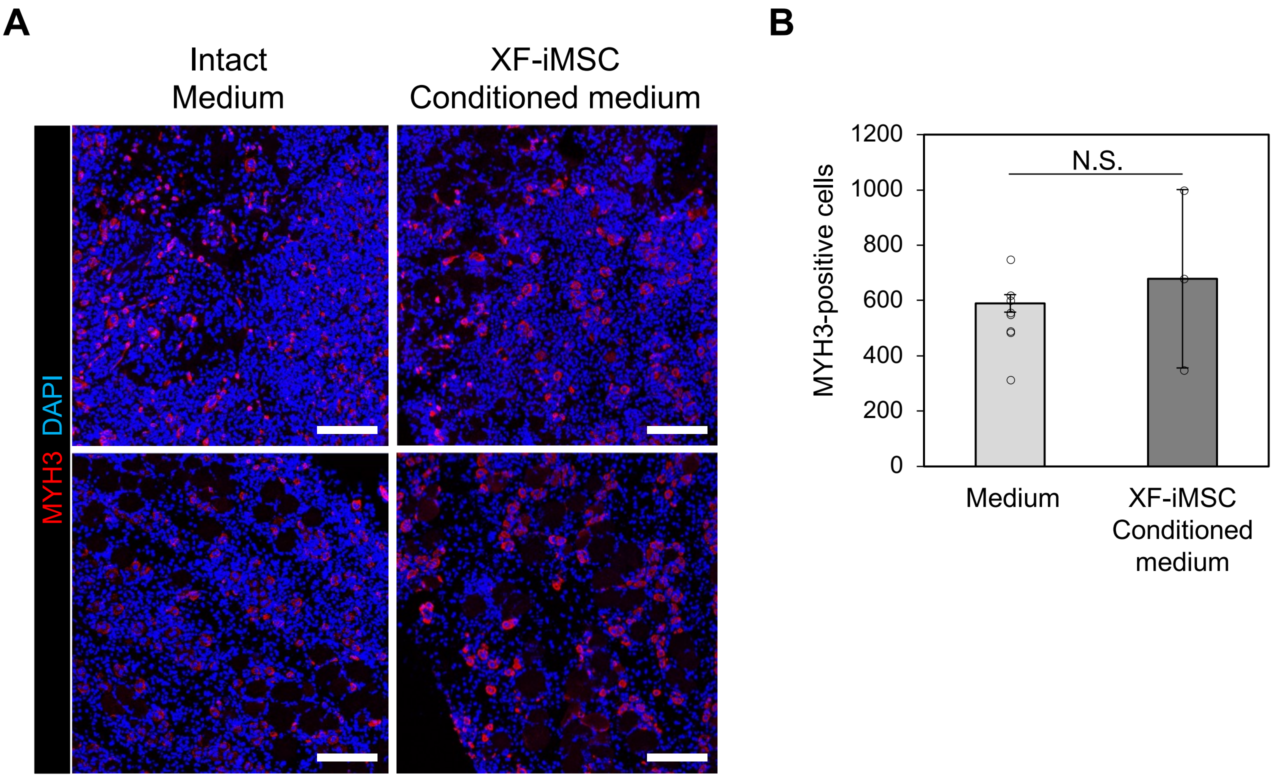

Supplementary Figure 12. Injection of conditioned medium from XF-iMSCs into crushed skeletal muscle.

(A) Sectional images of injured TA muscle three days after intact medium (left column) or XF-iMSC-conditioned medium (right column) injection. Sections were stained with anti-MYH3 antibody (red). Nuclei were stained with DAPI (blue). Scale bars, 100 μm. (B) Numbers of MYH3-positive cells three days after medium injection. Data are mean ± SD n = 3. n.s.: not significant.

Supplementary Figure 13

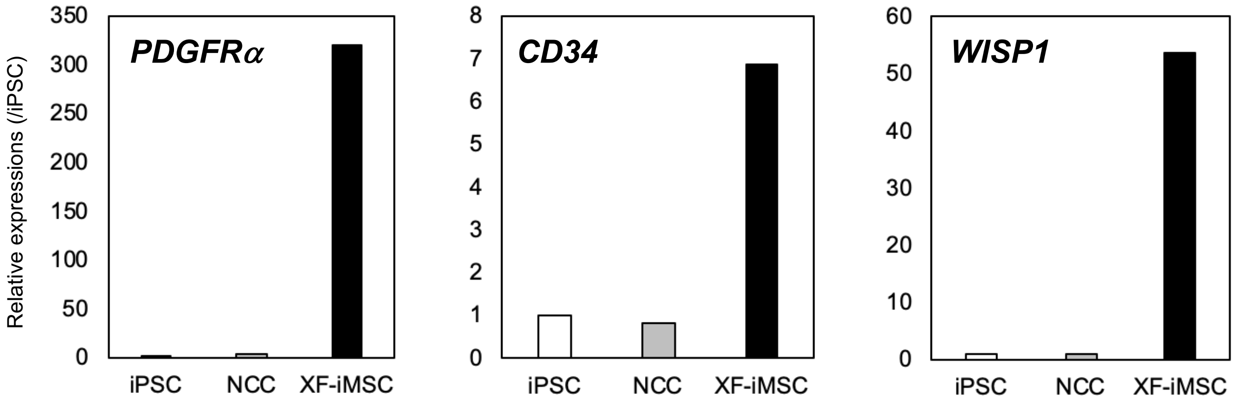

Supplementary Figure 13. XF-iMSC express fibro-adipogenic progenitor (FAP) marker genes. The expression of marker genes in 1231A3 iPSCs, NCCs, and XF-iMSCs. The mRNA expression of each gene was analyzed by the human transcriptome with Ion S5 XL. n = 2 (iPSC, NCC), n = 3 (XF-iMSC).

Supplementary Figure 14

A

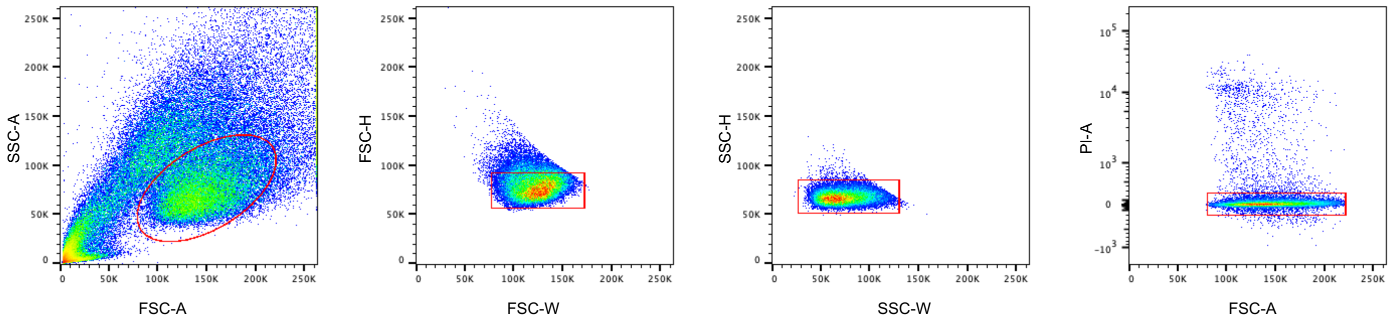

B

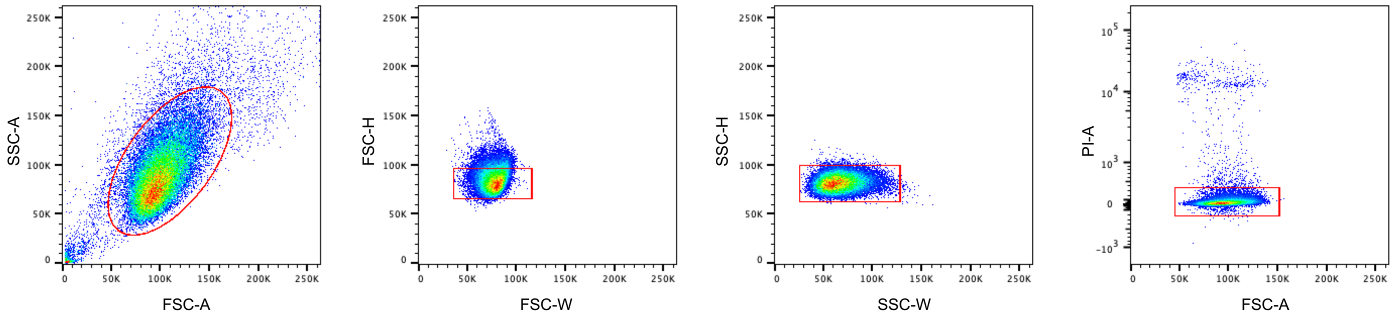

Supplementary Figure 14. FACS gating strategies

(A) Sequential gating (left to right panel) population of neural crest differentiation day 10 (Fig.1D and Supplementary Fig. 1B-E). Red circles indicated gating. Live cells were selected for PI-negative population (right panel). (B) Sequential gating (left to right panel) population of XF-iMSC PN4 (Fig.4D). Red circles indicated gating. Live cells were selected for PI-negative population (right panel).

Supplementary Table 1

Antibodies list

Antigen	Host	Clone	Conjugate	Source	Cat. No.	Dilution
SOX10	goat	poly	–	SantaCruz	sc-17342	1/200
CD271	mouse	mono	–	ATS	AB-N07	1/500
TFAP2A	mouse	mono	–	DSHB	3B5	1/500
TUBB3	rabbit	poly	–	abcam	ab18207	1/5000
Peripherin	mouse	mono	–	SantaCruz	sc-377093	1/200
GFAP	rabbit	poly	–	abcam	ab7260	1/500
MITF	rabbit	poly	–	Sigma	SAB4501879	1/200
TWIST	rabbit	poly	–	Merck	ABD29	1/500
DLX1	mouse	mono	–	NOVUS	H00001745-M03	1/200
hVimentin	rabbit	mono	–	abcam	ab16700	1/500
Laminin-α2	rat	mono	–	ALEXIS	ALX-804-190-C100	1/50
MYH4	mouse	mono	–	DSHB	BF-F3	1/50
MYH3	rabbit	poly	–	Sigma	HPA021808	1/200
h-Lamin A/C	mouse	mono	–	SantaCrutz	sc-7292	1/200
MHC	mouse	mono	–	R&D	MAB4470	1/50

Antigen	Host spec	Clone	Conjugate	Source	Cat. No.	Dilution
CD271	mouse	mono	Alexa647	BD Pharmingen	560326	1/100
CD44	mouse	mono	APC	BD Pharmingen	559942	1/100
CD45	mouse	mono	APC	BD Pharmingen	560973	1/100
CD73	mouse	mono	APC	BD Pharmingen	560847	1/100
CD90	mouse	mono	APC	BD Pharmingen	559869	1/100
CD105	mouse	mono	APC	eBioscience	17-1057	1/100
HLA-DR	mouse	mono	APC	BD Pharmingen	340549	1/100
CD29	mouse	mono	APC	BD pharmingen	561794	1/100
CD34	mouse	mono	APC	BD pharmingen	560940	1/100
SSEA4	mouse	mono	PE	BD Pharmingen	560128	1/100
Mouse_IgG1_k	mouse	mono	Alexa647	BD Pharmingen	557714	1/100
Mouse_IgG2b_k	mouse	mono	APC	BD Pharmingen	555745	1/100
Mouse_IgG1_k	mouse	mono	APC	BD Pharmingen	555751	1/100
Mouse_IgG2a_k	mouse	mono	APC	BD Pharmingen	555576	1/100

Supplementary Table 2

qPCR primers list

Gene	sense	antisense
NGFR	CCGTTGGATTACACGGTCCA	GACAGGGATGAGGTTGTCGG
SOX10	GAGCTGGACCGCACACCTTGGG	AACGCCCACCTCCTCGGACCTC
TFAP2A	AGGGCCTCGGTGAGATAGTT	AAGAGTTCACCGACCTGCTG
RHOB	ATCCCCGAGAAGTGGGTCC	CGAGGTAGTCGTAGGCTTGGA
PAX3	CGGCATCCTGAGCGAGCGAG	ACTCGGGCCTCGGTGAGCTT
SNAI2	TGTGACAAGGAATATGTGAGCC	TGAGCCCTCAGATTTGACCTG
CDH6	CTGCGACGGATGCAGATGAT	CCCTGTTTTCTCGATCCATGTTG
PAX6	CTGGCTAGCGAAAAGCAACAG	CCCGTTCAACATCCTTAGTTTATCA
TWIST	GTCCGCAGTCTTACGAGGAG	GCTTGAGGGTCTGAATCTTGCT
DLX1	TGCCAGAAAGTCTCAACAGCC	CGAGTGTAAACAGTGCATGGA
CDH11	AGAGAGCCCAGTACACGTTGA	TTGGCATGATAGGTCTCGTGC
POU5F1	GACAGGGGGAGGGGAGGAGCTAGG	CTTCCCTCCAACCAGTTGCCCCAAAC
ALP	GCGGTGAACGAGAGAATG	CGTAGTTCTGCTCGTGCAC
OCN	GTGACGAGTTGGCTGACC	TGGAGAGGAGCAGAACTGG
RUNX2	ACTACCAGCCACCGAGACCA	ACTGCTTGCAGCCTTAAATGACTC
BMP2	CTGTATCGCAGGCACTCA	CTCCGTGGGGATAGAACTT
BMP4	CACAGCACTGGTCTTGAGTATCCTG	CTCAGGGATGCTGCTGAGGTTAAAG
BMP7	CCACCTGTAATCCCAGCACT	GACAAAGACCAGAGGGTCCA
18S	GTAACCCGTTGAACCCCATT	CCATCCAATCGGTAGTAGCG
ACTB	AGGTCTTTGCGGATGTCCACGT	CACCATTGGCAATGAGCGGTTC

Supplementary Movie

Supplementary Movie 1. Myoblasts cultured with PM for 3 days

Supplementary Movie 2. Myoblasts cultured with DM for 3 days

Supplementary Movie 3. Myoblasts cultured with XF-iMSC cultured DM for 3 days

Supplementary Movie 4. Myoblasts cultured with DM + PXDN for 3days
