# Supplementary material for: Induction of functional xeno-free MSCs from human iPSCs via a neural crest cell lineage
Source: NPJ Regen Med. 2022 Sep 15;7:47. doi: 10.1038/s41536-022-00241-8 (PMC9477888; doi:10.1038/s41536-022-00241-8)
Supplement: Supplementary file 6 — REPORTING SUMMARY [file 41536_2022_241_MOESM6_ESM.pdf]

Corresponding author(s): Makoto Ikeya
Last updated by author(s): Jul 21, 2022

Reporting Summary

Nature Portfolio wishes to improve the reproducibility of the work that we publish. This form provides structure for consistency and transparency in reporting. For further information on Nature Portfolio policies, see our Editorial Policies and the Editorial Policy Checklist.

Statistics

For all statistical analyses, confirm that the following items are present in the figure legend, table legend, main text, or Methods section.

n/a	Confirmed	
☐	☒	The exact sample size (n) for each experimental group/condition, given as a discrete number and unit of measurement
☐	☒	A statement on whether measurements were taken from distinct samples or whether the same sample was measured repeatedly
☐	☒	The statistical test(s) used AND whether they are one- or two-sided<br>*Only common tests should be described solely by name; describe more complex techniques in the Methods section.*
☒	☐	A description of all covariates tested
☒	☐	A description of any assumptions or corrections, such as tests of normality and adjustment for multiple comparisons
☐	☒	A full description of the statistical parameters including central tendency (e.g. means) or other basic estimates (e.g. regression coefficient) AND variation (e.g. standard deviation) or associated estimates of uncertainty (e.g. confidence intervals)
☐	☒	For null hypothesis testing, the test statistic (e.g. F, t, r) with confidence intervals, effect sizes, degrees of freedom and P value noted<br>*Give P values as exact values whenever suitable.*
☒	☐	For Bayesian analysis, information on the choice of priors and Markov chain Monte Carlo settings
☒	☐	For hierarchical and complex designs, identification of the appropriate level for tests and full reporting of outcomes
☒	☐	Estimates of effect sizes (e.g. Cohen's d, Pearson's r), indicating how they were calculated

Our web collection on statistics for biologists contains articles on many of the points above.

Software and code

Policy information about availability of computer code

Data collection	BZ-X700, Countess II FL, FACS AriaII, QuantStudio 7, Ion S5 XL, Nikon Eclipse E600 microscope, SkyScan1176 in vivo micro-CT, Zeiss LSM 510 and 710 laser scanning confocal microscope, Olympus BX51 microscope, Easy n-LC 1000 HPLC, Orbitrap fusion tribrid mass spectrometer,
Data analysis	QuantStudio 7 software, Hybrid cell count software BZ-H3C, ImageJ, Bruker CTVOL software, Bruker CT-An software, Mascot search, Microsoft Excel, R version 2.8.1, R Commander 1.4-8

For manuscripts utilizing custom algorithms or software that are central to the research but not yet described in published literature, software must be made available to editors and reviewers. We strongly encourage code deposition in a community repository (e.g. GitHub). See the Nature Portfolio guidelines for submitting code & software for further information.

Data

Policy information about availability of data

All manuscripts must include a data availability statement. This statement should provide the following information, where applicable:
- Accession codes, unique identifiers, or web links for publicly available datasets
- A description of any restrictions on data availability
- For clinical datasets or third party data, please ensure that the statement adheres to our policy

RNA-seq data that support the findings of this study have been deposited in the Gene Expression Omnibus (GEO) database with the following accession code

GSE206048, GSE206128, GSE206172. Proteome data that support the findings of this study have been deposited in Japan Proteome Standard Repository (jPOSTrepo) database with the following accession code JPST001693.

Human research participants

Policy information about studies involving human research participants and Sex and Gender in Research.

Reporting on sex and gender	n/a
Population characteristics	n/a
Recruitment	n/a
Ethics oversight	n/a

Note that full information on the approval of the study protocol must also be provided in the manuscript.

Field-specific reporting

Please select the one below that is the best fit for your research. If you are not sure, read the appropriate sections before making your selection.

☒ Life sciences ☐ Behavioural & social sciences ☐ Ecological, evolutionary & environmental sciences

For a reference copy of the document with all sections, see nature.com/documents/nr-reporting-summary-flat.pdf

Life sciences study design

All studies must disclose on these points even when the disclosure is negative.

Sample size	Required sample sizes were estimated based on established protocols in the research field. The sample sizes were adequate as the differences between experimental groups were reproducible. We ensured that addition of sample sizes does not significantly affect the statistical outcomes. All n values are reported in the figure legends.
Data exclusions	n/a
Replication	To ensure the reproducibility data, we performed all experiments several independent times. Exact number of n for each experiment was written in the figure legends. Each independent experiment contains technical triplicates. We confirmed that these independent datasets did not change the interpretation of results.
Randomization	n/a
Blinding	n/a

Reporting for specific materials, systems and methods

We require information from authors about some types of materials, experimental systems and methods used in many studies. Here, indicate whether each material, system or method listed is relevant to your study. If you are not sure if a list item applies to your research, read the appropriate section before selecting a response.

Materials & experimental systems

n/a	Involved in the study	
☐	☒	Antibodies
☐	☒	Eukaryotic cell lines
☒	☐	Palaeontology and archaeology
☐	☒	Animals and other organisms
☒	☐	Clinical data
☒	☐	Dual use research of concern

Methods

n/a	Involved in the study	
☒	☐	ChIP-seq
☐	☒	Flow cytometry
☒	☐	MRI-based neuroimaging

Antibodies

Antibodies used | SOX10: goat, SantaCruz, sc-17342; CD271: mouse, ATS, AB-N07; TFAP2A: mouse, DSHB, 3B5; TUBB3: rabbit, abcam, ab18207; Peripherin: mouse, SantaCruz, sc-377093; GFAP: rabbit, abcam, ab7260; MITF: rabbit, Sigma, SAB4501879; TWIST: rabbit, Merck, ABD29; DLX1: mouse, NOVUS, H00001745-M03; hVimentin: rabbit, abcam, ab16700; Laminin-a2: rat, ALEXIS, ALX-804-190-C100; MYH4: mouse, DSHB, BF-F3; MYH3: rabbit, Sigma, HPA021808; h-Lamin A/C: mouse, SantaCrutz, sc-7292; MHC: mouse, R&D, MAB4470; CD271: mouse, BD Pharmingen, 560326; CD44: mouse, BD Pharmingen, 559942; CD45: mouse, BD Pharmingen, 560973; CD73: mouse, BD Pharmingen, 560847; CD90: mouse, BD Pharmingen, 559869; CD105: mouse, eBioscience, 17-1057; HLA-DR: mouse,BD Pharmingen, 340549; CD29: mouse, BD Pharmingen, 561794; CD34: mouse, BD Pharmingen, 560940; SSEA: mouse, BD Pharmingen, 560128; Mouse_IgG1_k: mouse, BD Pharmingen, 557714; Mouse_IgG2b_k: mouse, BD Pharmingen, 555745; Mouse_IgG1_k: mouse, BD Pharmingen, 555751; Mouse_IgG2a_k: mouse, BD Pharmingen, 555576

Validation | All antibodies were validated by suppliers accordingly.

Eukaryotic cell lines

Policy information about cell lines and Sex and Gender in Research

Cell line source(s) | human iPS cell lines, 1231A3, 1381A5, 1381B5, 1383D2, and 1383D10, was established at Center for iPS Cell Research and Application, Kyoto University.

Authentication | Authentication was unnecessary due to the unique morphology and differentiation potential of the iPSCs.

Mycoplasma contamination | The cell line tested negative for mycoplasma contamination.

Commonly misidentified lines (See ICLAC register) | n/a

Animals and other research organisms

Policy information about studies involving animals; ARRIVE guidelines recommended for reporting animal research, and Sex and Gender in Research

Laboratory animals | Male NOD/SCID mice (7 to 8-week-old), 8 - to 16-week-old NSG mice, newborn C57BL/6 mice

Wild animals | n/a

Reporting on sex | n/a

Field-collected samples | n/a

Ethics oversight | Ethical approval for these experiments was obtained from the Animal Care Committee of Shonan Health Innovation Park, Kyoto University, and Hiroshima University.

Note that full information on the approval of the study protocol must also be provided in the manuscript.

Flow Cytometry

Plots

Confirm that:

☒ The axis labels state the marker and fluorochrome used (e.g. CD4-FITC).

☒ The axis scales are clearly visible. Include numbers along axes only for bottom left plot of group (a 'group' is an analysis of identical markers).

☒ All plots are contour plots with outliers or pseudocolor plots.

☒ A numerical value for number of cells or percentage (with statistics) is provided.

Methodology

Sample preparation | The cells were trypsinized (or harvested with Accutase), re-suspended in PBS, and stained with primary antibodies for 30 min at 4°C.

Instrument | BD AriaII

Software | FlowJo

Cell population abundance | Sorted samples were directly reanalyzed by flow cytometry. We confirmed that CD271high sort enriching CD271-positive cells at >99% purity.

Gating strategy

Gating strategies indicated in Supplementary Fig. 14.

☒ Tick this box to confirm that a figure exemplifying the gating strategy is provided in the Supplementary Information.
